# Cardiovascular Risk Through Hypoxic Burden in Children With Sleep Apnea: A Secondary Analysis of a Nonrandomized Clinical Trial

**DOI:** 10.1001/jamanetworkopen.2025.38744

**Published:** 2025-10-23

**Authors:** Olga Mediano, Sonia López-Monzoni, María Castillo-García, Sofía Romero-Peralta, María Esther Viejo-Ayuso, Laura Silgado-Martínez, Leticia Álvarez-Balado, Carles Forné, Pilar Resano-Barrio, Francisco García-Rio, Irene Cano-Pumarega, Manuel Sánchez-de-la-Torre, Alfonso Ortigado, Laura Fidalgo, Ángel Rodríguez, Ainhoa Álvarez, Jorge Ullate Mora, Belén García-Mediano, Ali Azarbarzin, Esther Solano-Pérez

**Affiliations:** 1 Sleep Unit, Pneumology Department, Hospital Universitario de Guadalajara, Guadalajara, Spain; 2 Centro de Investigación Biomédica en Red de Enfermedades Respiratorias, Madrid, Spain; 3 Instituto de Investigación Sanitaria Castilla La Mancha, Toledo, Spain; 4 Medicine Department, Universidad de Alcalá, Madrid, Spain; 5 Heorfy Consulting, Lleida, Spain; 6 Department of Basic Medical Sciences, University of Lleida, Lleida, Spain; 7 Pneumology Department, Hospital Universitario La Paz, Instituto de Investigación Hospital Universitario La Paz, Madrid, Spain; 8 Sleep Unit, Pneumology Department, Hospital Universitario Ramón y Cajal, Instituto Ramón y Cajal de Investigación Sanitaria, Madrid, Spain; 9 Department of Nursing, Physiotherapy and Occupational Therapy, Faculty of Physiotherapy and Nursing, Universidad de Castilla la Mancha, Toledo, Spain; 10 Paediatric Department, Hospital Universitario de Guadalajara, Guadalajara, Spain; 11 Otorhinolaryngology Department, Hospital Universitario de Guadalajara, Guadalajara, Spain; 12 Osakidetza Basque Health Service, Araba University Hospital, Sleep Unit, Vitoria-Gasteiz, Spain; 13 Instituto de Investigación Sanitaria Bioaraba, Neurosciences, Vitoria-Gasteiz, Spain; 14 Division of Sleep and Circadian Disorders, Brigham and Women’s Hospital, Harvard Medical School, Boston, Massachusetts

## Abstract

**Importance:**

Emerging biomarkers, such as hypoxic burden (HB), have demonstrated utility in estimation of cardiovascular (CV) risk in adults with obstructive sleep apnea (OSA). Its applicability in pediatric OSA remains unexplored.

**Objective:**

To explore whether HB is associated with disturbances in blood pressure (BP) patterns in pediatric OSA.

**Design, Setting, and Participants:**

Children with suspected OSA were included in this secondary analysis performed within the framework of the Kids Trial, a prospective, multicenter nonrandomized clinical trial, with data accrued between January 30, 2018, and August 28, 2023, at 2 university hospitals in Spain. A total of 286 children with suspected OSA were assessed for eligibility. Ninety-six children for whom HB was unavailable or with BP data that did not fulfill quality criteria were excluded.

**Main Outcomes and Measures:**

Children underwent polysomnography and 24-hour ambulatory BP monitoring (ABPM). HB was quantified as the area under the desaturation curve of each respiratory event, relative to the pre-event baseline oxygen saturation. BP variables were obtained from ABPM.

**Results:**

A total of 190 children were included in the analysis. Median age was 6 (IQR, 5-8) years, and 108 participants (56.8%) were male. Median apnea hypopnea index was 6.0 (IQR, 3.1-10.3) events per hour with a median HB of 9.6 (IQR, 3.8-22.5) %min/h. A comparison of the HB quartiles revealed that higher HB levels were associated with greater nocturnal diastolic BP (quartile 1: 56.0 [IQR, 52.0-60.0] mm Hg; quartile 2: 57.0 [IQR, 54.0-61.0] mm Hg; quartile 3: 59.0 [IQR, 53.0-61.0] mm Hg; and quartile 4: 58.0 [IQR, 55.0-61.0] mm Hg; *P* = .03), a reduced nocturnal decrease in mean BP (quartile 1: 13.5 [IQR, 8.0-18.2] mm Hg; quartile 2: 10.8 [IQR, 8.3-15.1] mm Hg; quartile 3: 11.4 [IQR, 8.7-15.2] mm Hg; and quartile 4: 8.9 [IQR, 6.6-13.5] mm Hg; *P* = .01), and an increased prevalence of a nondipping pattern (NDP) (quartile 1: 15 of 47 [31.9%]; quartile 2: 21 of 47 [44.7%]; quartile 3: 19 of 46 [41.3%]; and quartile 4: 26 of 47 [55.3%]; *P* = .04). Multivariable analysis showed an increased risk of NDP in the higher quartile of HB (odds ratio, 2.41; 95% CI, 1.00-5.79; *P* = .05).

**Conclusions and Relevance:**

In this secondary analysis of a nonrandomized clinical trial, elevated HB values were associated with an NDP and reduced nocturnal decrease in BP in children with OSA, both of which are established markers of CV risk. These results suggest the potential utility of HB as a biomarker for CV risk stratification and clinical management in the pediatric population with OSA.

## Key Points

**Question:**

Does hypoxic burden correlate with nocturnal blood pressure and the nondipping pattern in pediatric obstructive sleep apnea (OSA)?

**Findings:**

In this secondary analysis of a nonrandomized clinical trial that included 190 children with suspected OSA, elevated values of hypoxic burden were associated with a reduced decrease of nocturnal blood pressure and an increased risk of a nondipping pattern.

**Meaning:**

These findings suggest that hypoxic burden may be a potential marker of cardiovascular risk in the pediatric population with OSA.

## Teaser

This secondary analysis of a nonrandomized clinical trial evaluates the association between hypoxic burden and blood pressure patterns in children with obstructive sleep apnea in Spain.

## Introduction

Obstructive sleep apnea (OSA) is highly prevalent sleep-disordered breathing in children, often associated with significant comorbidities, particularly cardiovascular (CV) consequences.^1^ OSA results from recurrent upper airway obstruction, typically caused by adenotonsillar hypertrophy, leading to partial (hypopnea) or complete (apnea) obstruction. These events disrupt oxygenation, ventilation, and respiratory patterns, contributing to substantial physiological alterations.^2^

These recurrent events result in increased sympathetic activity, coagulable and inflammatory states, and oxidative stress leading to endothelial dysfunction.^3^ Children with OSA have been found to have greater blood pressure (BP) variability, elevated systolic BP (SBP) and diastolic BP (DBP), and reduced nocturnal BP dipping. These changes represent modifiable CV risk factors, potentially predisposing children to hypertension and CV diseases in adulthood. Identifying these patterns early is crucial for risk stratification and targeted intervention in pediatric OSA populations.^1^

Traditionally, the number of respiratory events per hour (apnea-hypopnea index [AHI]) has been used to assess the severity of the disease. However, AHI has well-known limitations in accurately capturing the full spectrum of OSA regarding clinical manifestations or associated risks. Several studies have questioned its utility in predicting CV disease because AHI only reflects the frequency of respiratory events without considering some aspects of the immediate consequences, such as intermittent hypoxia, that are key to the pathophysiology and development of CV risk.^2^ Consequently, alternative parameters, such as hypoxic burden (HB), have been explored to enhance risk stratification and treatment optimization in patients with OSA, particularly adults.

HB, introduced in 2019 by Azarbarzin et al,^4^ quantifies the cumulative impact of OSA-related hypoxemia by incorporating the frequency, duration, and depth of oxygen desaturation events. HB is obtained from the sleep study by adding all individual desaturation areas and dividing by the total sleep time, with the units of HB being %min/h. HB values and their behavior throughout the sleep cycles, including dynamics across rapid eye movement (REM) and non-REM phases, as well as variation with positional changes, remain largely unexplored in the pediatric population.

In adults, HB has shown a robust independent association with CV mortality and comorbidities, including hypertension,^5^ incident heart failure,^6,7,8^ and venous thromboembolism.^9^ Despite HB’s potential as a more precise tool for risk stratification and treatment assessment in adults, its applicability and clinical relevance in the pediatric populations remains unexplored.

Our study evaluated the association between HB and BP patterns in the pediatric population with OSA. By investigating HB in children, we seek to establish its potential as a predictive marker for CV risk, thereby contributing to improved risk stratification and clinical management in this vulnerable group. As a secondary objective, we studied the value of HB throughout sleep stages and body position as factors associated with BP alteration.

## Methods

### Study Population

The Kids Trial cohort is part of a longitudinal, prospective study whose protocol was previously published^10^ and registered.^11^ The Kids Trial was conducted at 2 university hospitals in Spain. Participants were enrolled in the study from sleep units of Hospital Universitario de Guadalajara, Guadalajara, and Hospital Universitario de Araba, Vitoria-Gasteiz, when systematically referred for suspected OSA. Exclusion criteria consisted of (1) associated comorbidities, including CV or cerebrovascular disease and unstable severe or exacerbated respiratory disease; (2) genetic syndromes; (3) chronic insomnia and/or depressive syndrome; (4) history of otolaryngologic surgery or prior treatment with continuous positive airway pressure; and (5) contraindications for ambulatory BP monitoring (ABPM), including arrhythmias, latex allergy, or coagulation disorders.

### Ethics

This study was reported in accordance with the Transparent Reporting of Evaluations With Nonrandomized Designs (TREND) guidelines for nonrandomized clinical trials. The study adhered to the Declaration of Helsinki^12^ and was approved by the Ethics and Clinical Trials Committee of Hospital Universitario de Guadalajara. For children younger than 12 years, parents signed the informed consent; for children were 12 years or older, both parents and children signed the informed consent. A specific informed consent was signed for subclinical organ damage determination. The trial protocol and statistical analysis plan are found in Supplement 1.

### Clinical Assessment

Data were accrued between January 30, 2018, and August 28, 2023. Baseline clinical evaluations included the Pediatric Sleep Questionnaire, a validated tool completed by parents to assess the child’s sleep symptoms and wake behaviors. The physical examination included a series of anthropometric measurements, such as weight, height, and body mass index (BMI), as well as measurements of the neck, hip, and waist circumference. Upper airway assessments included evaluations of microretrognathia, Mallampati classification, adenotonsillar hypertrophy, ogival palate, and bite alignment. BMI percentiles were used to stratify participants as having underweight (<5th percentile), normal weight (5th-85th percentile), overweight (85th-95th percentile), or obesity (>95th percentile).

### Sleep Study

Polysomnography (PSG) was performed and scored following the standards set by the 2017 update of the American Academy of Sleep Medicine scoring manual, version 2.4.^13^ Nasal flow, snoring, thermistor, thoracic and abdominal movement, electrocardiography, oxygen saturation, transcutaneous capnography, body position, and leg movement signals were recorded. The electroencephalogram included recordings of 6 electrodes referred to in accordance with the 10-20 rules of the international system, including also electromyographic and electro-oculographic signals. Apnea was defined as a reduction in airflow of greater than 90% during 2 respiratory cycles in the case of obstructive apnea or a period of greater than 20 seconds or 2 respiratory cycles accompanied by desaturation of 3% or a microarousal in the case of central apnea. Hypopnea was defined as a decrease in airflow of 30% to 90% during 2 respiratory cycles, accompanied by a desaturation of greater than 3% or a microarousal. A valid PSG (good-quality study) was defined as having at least 300 minutes of study and at least 180 minutes of sleep. Diagnosis of OSA followed the current guidelines.^14^ AHI was calculated as the total number of respiratory events divided by the number of hours of sleep.

### HB Assessment

HB was defined as the total area under the respiratory event–related desaturation curve. This value was calculated as described by Azarbarzin et al,^4^ whereby the sum of all individual desaturation areas was divided by the total sleep time to yield a result expressed as %min/h.^4^ The specific HB quantifies the total amount of hypoxemia associated with respiratory events. The algorithm was implemented using a diagnostic sleep system (Alice 6 LDxS; Philips Respironics), and Sleepware software, version 4.0.1.0 (Koninklijke Philips NV). Specific HB throughout sleep stages (REM and non-REM) and body position (supine and nonsupine) was also calculated. HB was categorized into quartiles based on its empirical distribution.

### BP Determinations

Clinic BP measurements were performed using a pediatric-validated sphygmomanometer on the nondominant arm after 5 minutes of rest, with the child seated, legs uncrossed, and bladder voided. Three measurements were taken at 3-minute intervals, with the mean of the last 2 readings used for analysis.

ABPM was conducted using a monitor validated for pediatric populations (OnTrak; Spacelabs), following the European Society of Hypertension guide for the management of hypertension in children.^15^ Measurements were taken every 20 minutes until 10 pm and every 30 minutes at night, with pediatric-sized cuffs adjusted to the child’s arm. A study was deemed interpretable and of good quality when it exhibited a minimum of 1 reading per hour, a total of 40 successful readings, and a success rate of at least 65% for the programmed readings. The reported times of waking up and going to sleep were used to define day and night periods. Children with mean SBP and/or DBP of at least the 90th but less than the 95th percentile were classified as having high-normal BP (prehypertension). Hypertension was defined as persistently elevated SBP and/or DBP greater than the 95th percentile, adjusted for sex, age, and height. In the context of a dipping pattern, a decrease in SBP and DBP of at least 10% during the daytime vs the sleep period was considered within the normal range. A decrease in BP of less than 10% was considered indicative of a nondipping pattern (NDP).

### Statistical Analysis

This is a post hoc analysis of baseline data from the ongoing Kids Trial. Therefore, no formal sample size calculation was performed for the present analysis.

Categorical variables were described using frequencies and percentages, and continuous variables were summarized using the median (IQR [25th and 75th percentiles]), given their nonnormal distribution, as assessed by the Shapiro-Wilk test. Associations between study variables and OSA severity (based on AHI categories) or HB groups (based on sample quartiles) were examined using the Mantel-Haenszel test for trend for ordinal variables and the Spearman rank correlation test for continuous variables. Further, characteristics of included and excluded participants were compared using the Pearson χ^2^ test for qualitative variables and the Mann-Whitney test for quantitative variables, to assess potential selection bias.

Multivariable logistic regression models were used to assess the association between HB quartiles and the NDP, adjusting for sex, age, and BMI. Odds ratios (ORs) and 95% CIs were reported and illustrated with forest plots. For comparison purposes, an analogous model was fitted using AHI categories instead of HB. The discriminative performance of both models was assessed using the area under the receiver operating characteristic curve (AUROC), and AUROCs were compared using the DeLong test.

Additional multivariable logistic regression models were fitted to examine whether the association between HB and NDP was modified by overall SBP or DBP. Interaction terms between HB quartiles and BP variables were evaluated using likelihood ratio tests.

All hypothesis tests were 2 sided, with a significance threshold of less than .05. All analyses were conducted using R software, version 4.3.1 (R Program for Statistical Computing).

## Results

### Patient General Characteristics

**Figure 1.**
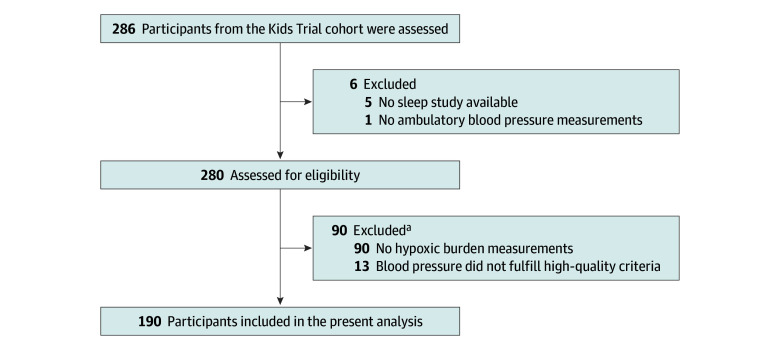
Trial Flow Diagram ^a^Some participants were excluded for both reasons.

A total of 286 children with suspected OSA were included in the Kids Trial study. Participants with good-quality PSG and ABPM studies and the possibility of HB calculation were included in the present analysis (Figure 1). The final population consisted of 190 children (82 [43.2%] female and 108 [56.8%] male), with a median age of 6 (IQR, 5-8) years (Table). Comparative data between excluded and included participants are presented in eTable 1 in Supplement 2. Anthropometric characteristics showed a median BMI at the 43.5th (IQR, 24.2th-77.8th) percentile. The median office SBP was 100 (IQR, 95-109) mm Hg, and the median office DBP was 66 (IQR, 61-72) mm Hg. The PSQ reading was positive in 112 participants (58.9%). Tonsillar hypertrophy (grade 3 tonsil size [51%-75%]) was the most frequent abnormality, observed in 86 of the 188 children with data available (45.7%). Additionally, 103 of 185 children with data available (55.7%) had a Mallampati class 2 of a possible 4.

**Table.** Characteristics of the Whole Study Population and by OSA Severity

Characteristic	OSA severity quartile					*P* value^a^
	Global (N = 190)	1 (n = 48)	2 (n = 48)	3 (n = 46)	4 (n = 48)	
**Demographic characteristics**						
Sex, No. (%)						
Female	82 (43.2)	22 (45.8)	19 (39.6)	19 (41.3)	22 (45.8)	.96
Male	108 (56.8)	26 (54.2)	29 (60.4)	27 (58.7)	26 (54.2)	
Age, median (IQR), y	6 (5-8)	7 (5-9)	5 (4-6)	6 (5-9)	5 (4-7)	.04
**Anthropometric characteristics**						
BMI percentile, median (IQR)	43.5 (24.2-77.8)	43.5 (27.5-87.5)	44.5 (24.0-77.0)	49.5 (30.5-81.8)	36.5 (21.8-67.2)	.40
Neck circumference, median (IQR), cm	26.5 (25.0-28.0)	27.0 (26.0-28.2)	26.5 (25.4-27.2)	27.0 (25.5-29.0)	26.0 (24.4-27.1)	.04
Waist circumference, median (IQR), cm	57.0 (53.0-65.0)	58.5 (54.8-66.5)	55.5 (53.4-63.2)	59.8 (53.2-66.8)	55.5 (51.0-60.0)	.14
Hip circumference, median (IQR), cm	63.0 (59.0-72.0)	66.0 (60.0-77.0)	63.0 (59.0-68.2)	66.0 (59.0-75.2)	61.0 (57.0-66.2)	.04
SBP, median (IQR), mm Hg	100 (95-109)	102 (94-109)	100 (94-109)	101 (95-109)	100 (94-107)	.53
DBP, median (IQR), mm Hg	66 (61-72)	66 (61-70)	64 (59-69)	68 (60-73)	67 (64-73)	.18
PSQ positive (abnormal), No. (%)	112 (58.9)	25 (52.1)	27 (56.2)	28 (60.9)	32 (66.7)	.13
**Otorhinolaryngology physical examination**						
Mallampati classification, No. (%)^b^						
1	41 (22.2)	10 (21.3)	8 (16.7)	9 (19.6)	14 (31.8)	.11
2	103 (55.7)	27 (57.4)	26 (54.2)	24 (52.2)	26 (59.1)	
3	41 (22.2)	10 (21.3)	14 (29.2)	13 (28.3)	4 (9.1)	
4	0	0	0	0	0	
Tonsil size, No. (%)^b^						
Grade 1 (0%-25%)	5 (2.7)	2 (4.2)	1 (2.1)	0	2 (4.3)	.07
Grade 2 (26%-50%)	40 (21.3)	13 (27.1)	10 (20.8)	11 (23.9)	6 (13.0)	
Grade 3 (51%-75%)	86 (45.7)	24 (50.0)	20 (41.7)	21 (45.7)	21 (45.7)	
Grade 4 (76%-100%)	57 (30.3)	9 (18.8)	17 (35.4)	14 (30.4)	17 (37.0)	
Palate (ogival), No. (%)^b^	71 (37.6)	16 (33.3)	16 (34.0)	20 (43.5)	19 (39.6)	.37
Mandible (abnormal), No. (%)	45 (23.7)	14 (29.2)	11 (22.9)	9 (19.6)	11 (22.9)	.43
Bite, No (%)^b^						
Class 3	17 (23.3)	5 (21.7)	3 (16.7)	6 (33.3)	3 (21.4)	.18
Class 2	47 (64.4)	13 (56.5)	13 (72.2)	10 (55.6)	11 (78.6)	
Open	9 (12.3)	5 (21.7)	2 (11.1)	2 (11.1)	0	

Abbreviations: BMI, body mass index calculated as weight in kilograms divided by height in meters squared); DBP, diastolic blood pressure; OSA, obstructive sleep apnea; PSQ, Pediatric Sleep Questionnaire; SBP, systolic blood pressure.

^a^ Spearman rank correlation test for continuous variables and the Mantel-Haenszel test for trend for categorial variables were used.

^b^ Owing to missing data, numbers may not sum to totals in column heading.

### HB Parameters in the Pediatric Population With OSA

**Figure 2.**
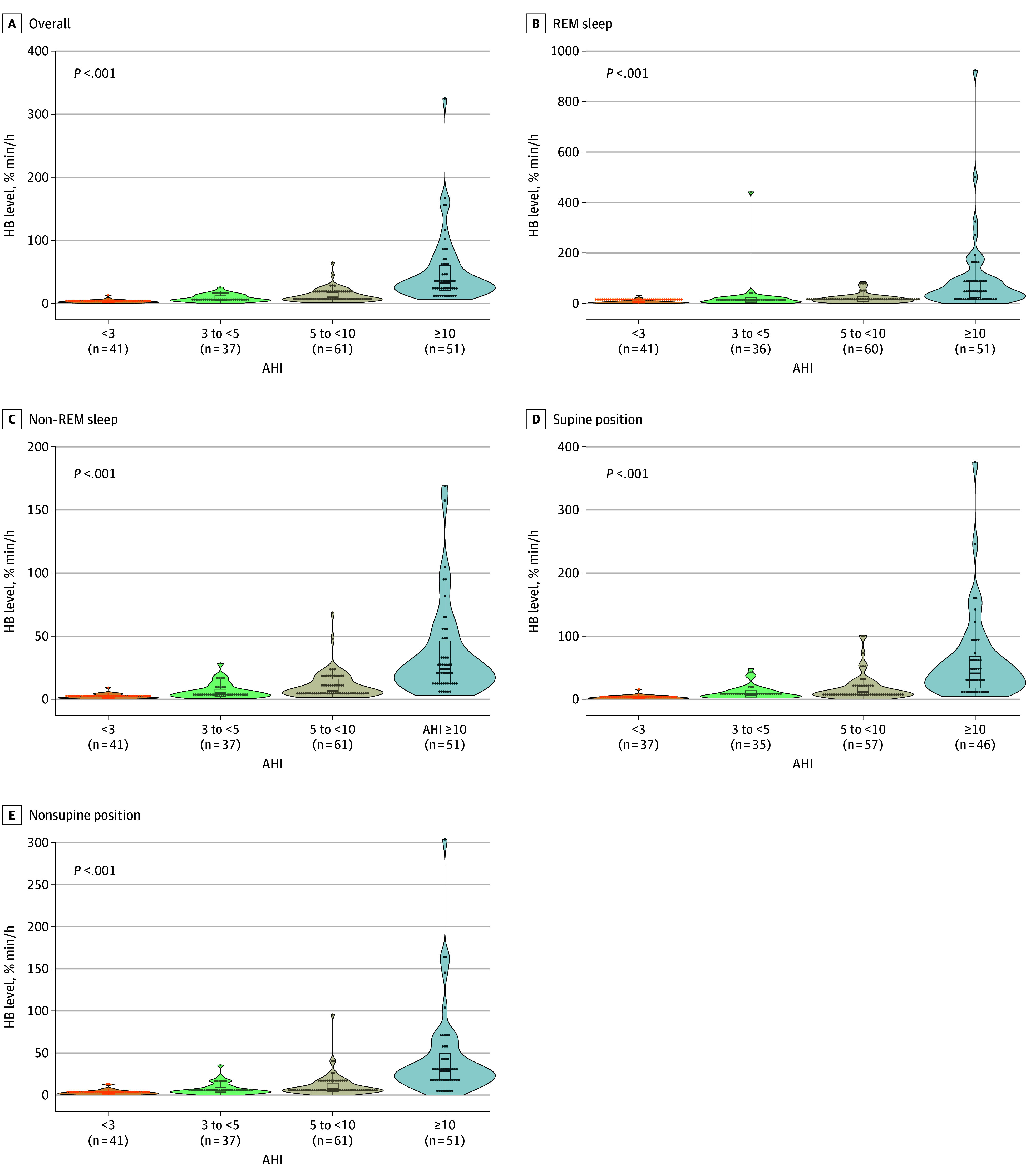
Measures of Hypoxic Burden (HB) for Different Sleep Stages and Positions as a Function of Obstructive Sleep Apnea (OSA) Severity in Pediatric Patients Spearman rank correlation test was used. AHI indicates apnea-hypopnea index (calculated as the number of respiratory events per hour); REM, rapid eye movement.

The median AHI was 6.0 (IQR, 3.10-10.3) events per hour with a mean oxygen saturation (Spo_2_) of 96% (IQR, 95%-97%), minimum Spo_2_ of 89% (IQR, 86%-91%), and desaturation index of 5.3 (IQR, 3.2-10.5) events per hour (eTable 2 in Supplement 2). In total, 167 of 187 children (89.3%) presented with an obstructive AHI of at least 1/h. The median value of HB (%min/h) in pediatric patients was 9.6 (IQR, 3.8-22.5) %min/h. A progressive increase in HB values was observed with greater OSA severity, as classified by the AHI (Figure 2). Overall, mean (SD) HB for AHI of less than 3 was 3.1 (2.3) %min/h (median, 2.9 [IQR, 1.4-4.1] %min/h). Mean (SD) HB for AHI of 3 to less than 5 was 17.8 (57.0) %min/h (median, 6.6 [IQR, 4.4-13.5] %min/h). Mean (SD) HB for AHI of 5 to less than 10 was 12.8 (11.0) %min/h and median, 9.6 (IQR, 5.4-17.0) %min/h. Mean (SD) HB for AHI of 10 or greater was 50.5 (54.8) %min/h (median, 31.8 [IQR, 20.2-60.3] %min/h). Additional measurements were obtained for different sleep stages (REM and non-REM) and positions (supine and nonsupine), with similar results.

### HB and 24-Hour Ambulatory BP Parameters

Daytime BP medians were 78.0 (IQR, 74.0-81.0) mm Hg for mean BP, 102.0 (IQR, 96.0-107.0) mm Hg for SBP, and 68.0 (IQR, 64.0-71.0) mm Hg for DBP. Nocturnal BP medians were 69.0 (IQR, 64.0-73.0) mm Hg for mean BP, 90.0 (IQR, 84.0-99.0) mm Hg for SBP, and 57.0 (IQR, 53.0-61.0) mm Hg for DBP. The median decrease in overall mean BP was 11.2 (IQR, 7.6-15.5) mm Hg. An NDP was observed in 81 of 187 patients with data available (43.3%). In addition, among 181 patients with data available, 3 (1.7%) were diagnosed with hypertension and 5 (2.8%) had high-normal BP (eTable 3 in Supplement 2).

When variables obtained from the ABPM were analyzed across quartile-based groups of HB, significant patterns were identified. A comparison of the HB quartiles revealed that higher HB levels were significantly associated with greater nocturnal DBP (quartile 1: 56.0 [IQR, 52.0-60.0] mm Hg; quartile 2: 57.0 [IQR, 54.0-61.0] mm Hg; quartile 3: 59.0 [IQR, 53.0-61.0] mm Hg; and quartile 4: 58.0 [IQR, 55.0-61.0] mm Hg; *P* = .03), a reduced nocturnal decrease in mean BP (quartile 1: 13.5 [IQR, 8.0-18.2] mm Hg; quartile 2: 10.8 [IQR, 8.3-15.1] mm Hg; quartile 3: 11.4 [IQR, 8.7-15.2] mm Hg; and quartile 4: 8.9 [IQR, 6.6-13.5] mm Hg; *P* = .01), and an increased prevalence of NDP (quartile 1: 15 of 47 [31.9%]; quartile 2: 21 of 47 [44.7%]; quartile 3: 19 of 46 [41.3%]; and quartile 4: 26 of 47 [55.3%]; *P* = .04).

When analyzed across additional sleep parameters, similar associations were observed between HB and ABPM outcomes. Specifically, for HB REM sleep, significant differences were found in nocturnal DBP, the overall mean BP decrease, and the prevalence of NDP. For HB non-REM sleep, no significant differences were identified. For HB supine position, associations were observed with nocturnal DBP, nocturnal mean BP, overall mean BP decrease, and NDP, while for HB in nonsupine position, associations were noted with nocturnal DBP, overall mean BP decrease, and NDP. Detailed results are provided in eTables 4 to 7 in Supplement 2).

**Figure 3.**
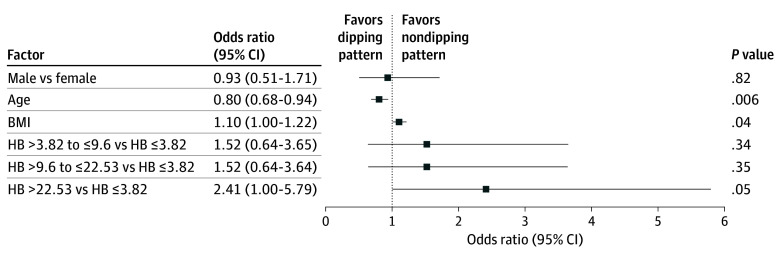
Forest Plot Showing the Result of the Multivariable Logistic Regression Analysis for Nondipping Pattern According to Hypoxic Burden (HB) Levels Odds ratios are adjusted for sex, age, and body mass index (BMI; calculated as the weight in kilograms divided by the height in meters squared).

After adjusting for sex, age, and BMI, children with HB values greater than 22.53 (%min/h) demonstrated a higher risk (OR, 2.41; 95% CI, 1.00-5.79; *P* = .05) of exhibiting NDP compared with those with lower HB values (Figure 3). These findings were consistent across REM (OR, 3.06; 95% CI, 1.24-7.54; *P* = .02) and non-REM (OR, 1.58; 95% CI, 0.68-3.67; *P* = .29) sleep as well as supine (OR, 2.75; 95% CI, 1.12-6.74; *P* = .03) and nonsupine (OR, 2.39; 95% CI, 1.01-5.62; *P* = .05) positions, as detailed in eFigures 1 to 4 in Supplement 2.

The AUROC was calculated to assess the discriminative ability of HB to identify NDP, yielding a value of 0.671 (95% CI, 0.593-0.749). The same analysis using AHI yielded an AUROC of 0.689 (95% CI, 0.611-0.766). A statistical comparison of the 2 AUROCs showed a difference of −0.018 (95% CI, −0.066 to 0.030; *P* = .46).

## Discussion

This secondary analysis of a nonrandomized clinical trial advances our understanding of the role of HB as a factor associated with CV risk in pediatric patients with OSA. Traditionally, the AHI has been the primary diagnostic tool for assessing OSA severity, although recent research has raised concerns about its sensitivity in reliably estimating CV risk, particularly in adults.

Using data from the Kids Trial cohort, this study provides measurement of HB in children with OSA for the first time. Our findings demonstrate that HB severity is associated with BP metrics and the presence of an NDP, a recognized marker of CV risk. Although HB has been extensively studied in adults, data on its utility in pediatric populations remain scarce. Given that children typically experience fewer and less severe desaturation events compared with adults, it is expected that the median HB values reported in this study are considerably lower than those observed in adult populations.^4^ Additionally, our findings highlight that HB values are influenced by specific sleep stages, such as REM, and body positions, particularly supine, with higher HB values observed in these contexts. To the best of our knowledge, this is the first study to demonstrate the association of sleep stage and position with HB values measured by PSG.

In adult populations, OSA has been consistently associated with elevated SBP, particularly during the night, which is thought to be driven by increased sympathetic activity and vascular stiffness.^16^ However, in our pediatric cohort, the most notable alterations were observed in nocturnal DBP. This difference may reflect developmental variations in vascular structure and autonomic regulation between children and adults. In children, peripheral vascular resistance plays a more dominant role in determining DBP, in which OSA may preferentially affect these mechanisms.^1^ Our findings suggest that DBP may be a more sensitive marker of early CV alterations related to OSA.

In terms of CV risk estimation, HB has been associated with both mortality and morbidity in diverse populations. Azarbarzin et al^4^ and Trzepizur et al^17^ reported that higher HB values, particularly those with an HB greater than 60 %min/h, were associated with heightened risk for CV morbidity and mortality.^18^ In our pediatric population, HB greater than 22.53 %min/h was associated with an NDP and a smaller nocturnal decrease in global mean BP, emphasizing the clinical relevance of HB in estimating CV outcomes in children with OSA.

The results of this study are consistent with those previously reported by our group,^19^ which demonstrated an association between BP levels and the AHI, a classic OSA severity parameter. These results showed that OSA severity was associated with a higher risk of NDP, which directly affected the circadian rhythm of BP. Similarly, our current results indicate that HB serves as a reliable marker of CV risk in this population.

Furthermore, our results suggest that the discriminative ability of HB in identifying NDP is comparable to that of the AHI. A possible explanation is that children tend to experience fewer and less pronounced oxygen desaturations, which may attenuate the contribution of hypoxia to the HB calculation. In contrast, the AHI also captures respiratory events associated with arousals, which might better reflect the autonomic disturbances contributing to BP alterations in this population.

A notable advantage of using HB is the simplicity and automation of its calculation, as it is derived from pulse oximetry signals and can be fully captured without the need for scoring respiratory events and sleep stages.^20,21^ This approach is particularly beneficial in pediatric populations, among whom a streamlined and cost-effective tool could facilitate timely treatment decisions aimed at reducing future CV risk. This in turn would reduce the burden on children who are on waiting lists. Identifying patients with a potential NDP is particularly important, as the night-day BP ratio and dipping pattern significantly and independently predict mortality and CV events in adults.^22^ Less information is available on what happens in children, but there is evidence that moderate-to-severe childhood OSA is associated with a higher risk of adverse SBP outcomes.^23^ Then, early identification and OSA treatment may promote CV in future adulthood. This study marks the first step toward tailoring therapeutic interventions in children to mitigate CV risk associated with OSA. Establishing a specific HB threshold for high-risk patients could also optimize resource utilization in diagnosing hypertension, as 24-hour BP monitoring poses technical and logistical challenges in pediatric populations.

The association between intermittent hypoxia and NDP is mediated by several pathophysiological mechanisms that increase long-term CV risk. The sympathetic nervous system is activated, disrupting its circadian regulation and leading to inadequate nocturnal BP reduction. In addition, sleep fragmentation and elevated cortisol levels further stimulate the renin-angiotensin-aldosterone system, promoting arterial stiffness and left ventricular hypertrophy. In addition, repeated fluctuations in oxygen levels induce oxidative stress, causing endothelial damage and perpetuating a chronic low-grade inflammatory state that contributes to endothelial dysfunction and the progression of atherosclerosis. These interrelated processes create a milieu of vascular dysfunction, arterial stiffness, and cardiac remodeling that significantly increases the likelihood of future CV events, underscoring the need for early detection and intervention in affected individuals.

This study provides HB values across various sleep stages, including REM and non-REM, as well as different sleep positions, such as supine and nonsupine, in children with OSA. This comprehensive approach is crucial for understanding how HB levels fluctuate under different sleep conditions and is consistent with those from adult populations in which OSA hypoxemia is worse in the supine position and REM sleep due to collapsibility and reduced upper airway muscle activity. Additionally, the integration of complete PSG and 24-hour ABPM significantly enhances the accuracy and reliability of diagnosing OSA and evaluating CV health. Together, these methods provide compelling and objective data on both sleep-related and CV factors, emphasizing the importance of rigorous assessment in this field.

### Limitations

This study has some limitations. First, HB was categorized using empirical quartiles. While this approach is useful for exploring associations across the distribution of values, it may limit the generalizability of the findings. However, this approach was chosen because, to our knowledge, this is the first study to describe and analyze this variable in the pediatric population. Second, although the study is based on a prospective cohort, the present analysis is cross-sectional, which limits the ability to infer causality between HB and CV outcomes. No formal sample size calculation was performed specifically for this analysis because it was a post hoc analysis of baseline data from an ongoing prospective study originally designed with a different primary objective (the Kids Trial study). Third, although the analyses were adjusted for key covariates, the possibility of residual confounding remains. Unmeasured variables could partly account for the observed association between high HB and CV risk. This study is based on data from a single cohort, which may restrict the generalizability of our findings. Nonetheless, this study addresses an important gap in the literature by evaluating HB as a promising CV risk biomarker in children with OSA. Our findings contribute critical insights into the value of HB specifically within the pediatric population, an area previously underexplored. However, validation in larger, more diverse populations is necessary.

## Conclusions

In this secondary analysis of a nonrandomized clinical study of a pediatric population with OSA, our findings suggest the potential utility of HB for CV risk stratification. Higher HB values were associated with NDP and a smaller decrease in global mean BP. These results identified HB as an emerging biomarker of CV risk in children, with potential implications for improving clinical practices in OSA management and guiding personalized therapeutic interventions in this population.

## Notes

Supplement 1.
Trial Protocol and Statistical Analysis Plan

Supplement 2. **eTable 1.** Comparison Between the Excluded Individuals and Those Included in the Analysis **eTable 2.** Polysomnographic Variables **eTable 3.** Measures of the Ambulatory 24-Hour Blood Pressure Parameters According to Quartile-Based Groups of HB **eTable 4.** Measures of the Ambulatory 24-Hour Blood Pressure Parameters According to the Hypoxic Burden in Rapid Eye Movement Sleep **eTable 5.** Measures of the Ambulatory 24-Hour Blood Pressure Parameters According to the Hypoxic Burden in Non–Rapid Eye Movement Sleep **eTable 6.** Measures of the Ambulatory 24-Hour Blood Pressure Parameters According to the Hypoxic Burden in the Supine Position **eTable 7.** Measures of the Ambulatory 24-Hour Blood Pressure Parameters According to the Hypoxic Burden in the Nonsupine Position **eFigure 1.** Forest plot Showing the Result of the Multivariable Logistic Regression Analysis for Nondipper Pattern According to HB in REM Sleep **eFigure 2.** Forest plot Showing the Result of the Multivariable Logistic Regression Analysis for Nondipper Pattern According to HB in non-REM Sleep **eFigure 3.** Forest Plot Showing the Result of the Multivariable Logistic Regression Analysis for Nondipper Pattern According to HB in Supine Position **eFigure 4.** Forest Plot Showing the Result of the Multivariable Logistic Regression Analysis for Nondipper Pattern According to HB in Nonsupine Position

Supplement 3.
Data Sharing Statement
