# Supplementary material for: Cardiovascular Risk Through Hypoxic Burden in Children With Sleep Apnea: A Secondary Analysis of a Nonrandomized Clinical Trial
Source: JAMA Netw Open. 2025 Oct 23;8(10):e2538744. doi: 10.1001/jamanetworkopen.2025.38744 (PMC12550637; doi:10.1001/jamanetworkopen.2025.38744)
Supplement: Supplement 1. — Trial Protocol and Statistical Analysis Plan [file jamanetwopen-e2538744-s001.pdf]

Prevalence of High Blood Pressure in Pediatric Patients with Sleep-Disordered Breathing. Reversibility after Treatment: The KIDS TRIAL Study Protocol

1. Introduction

1.1. Obstructive Sleep Apnea in Children

Obstructive sleep apnea (OSA) is the maximum expression of sleep-disordered breathing (SDB), ranging from simple snoring to OSA [1]. Pediatric OSA is defined as a breathing alteration during sleep, characterized by total (apnea) or partial (hypopnea) obstructions of the upper airway that interfere with normal ventilation and sleep architecture [2]. The severity of OSA is measured by the apnea–hypopnea index (AHI), which shows the number of respiratory events per hour during the sleep. OSA in children is a major public health problem given its high prevalence and its association with relevant consequences, fundamentally in the metabolic, neurocognitive, and cardiovascular spheres [3]. OSA children present nocturnal symptoms such as snoring, nocturnal apneas evidenced by the parents, and enuresis. During the day, the sleep apnea children present with hyperactivity, concentration and memory problems, and somnolence or tiredness [4]. Studies conducted on the prevalence of OSA in children have shown highly variable ratios depending on the population under study, the diagnostic method, and the definitions used. Previous studies have suggested a prevalence of OSA between 1–5% [4,5] in children, with adeno-tonsillar hypertrophy being the most common factor for developing OSA in childhood. A complete polysomnography (PSG) is the diagnostic test for OSA in children that is considered the "gold standard". Additionally, simplified methods have also been validated in the pediatric population [4].

Although being the same disease, OSA is very different between adults and children: the definitions for respiratory event and OSA severity are distinct, diagnosis tools used in adults have less utility in children, and treatment is usually definitive in resolving the events in children and not in adults.

1.2. OSA and Cardiovascular Consequences

During sleep, the patient with OSA is repeatedly subjected to intermittent hypoxia, changes in intrathoracic pressure, and microarousals, which results in the hyperactivity of the sympathetic nervous system, higher oxidative stress, and a proinflammatory and hypercoagulable state [6]. Several studies have suggested that these repeated alterations in each episode of apnea/hypopnea contribute to the development and progression of different cardiovascular diseases, well-documented for high blood pressure (HBP), contributing in these patients to higher mortality and morbidity. In adult patients with OSA, there is an increase in blood pressure (BP) and worse control of it. It has been shown that OSA patients suffer more frequently from a non-dipping pattern and resistant hypertension. The most consistent results relate treatment with continuous positive airway pressure

(CPAP) to a reduction in arterial BP [7] and the first signs of atherosclerosis [8]. Randomized clinical trials demonstrate that CPAP treatment reduced blood pressure, was more consistent in resistant hypertension patients and restored the non-dipping pattern [7]. An important aspect is that, to get this effect, a good compliance (better if is more than 6 h per night) is needed [9]. However, the efficacy of CPAP in reducing cardiovascular events has not yet been proven [10]. Among the factors that might have influenced this are: (1) the associated cardiovascular illness in OSA adults could be irreversible if treating the disease in adulthood; (2) the presence of cardiovascular risk factors (hypertension, diabetes mellitus, dyslipidemia, etc.) that can act as confounding factors, and (3) the treatment of OSA with CPAP has mainly been suboptimal in this type of clinical trial. For these reasons, the pediatric population constitutes an ideal target group for confirming the impact of OSA on cardiovascular risk prevention: free of established disease and preexisting risk factors, and with effective treatment such as adeno-tonsillectomy. In this way, knowledge of the natural history of the disease would also be facilitated by studying the OSA pediatric population.

1.3. Blood Pressure in SDB in Children

Unlike adults, the diagnosis of HBP in children is based on the normal distribution of BP in healthy children and not on the morbidity and mortality associated with it. In this group, ambulatory BP monitoring (ABPM) during 24 h is more favorable than isolated office BP in predicting morbidity and mortality. ABPM offers advantages over isolated measurements since it allows us to study the variability of BP throughout the circadian cycle and it is more appropriate for predicting organ damage. The 2016 European Guideline for the management of high blood pressure in children [11] continues assuming, as reference values, those provided by the US Task Force [12], in which the values are distributed according to percentiles based on gender, age, and height. However, ABPM is complicated to perform in children, given the difficulty associated with this technique obtaining good quality results.

In children with high BP values, even when these are close to normal levels, it is possible to predict the development of hypertension when they become adults [13], cardiometabolic risk [14], and future coronary disease [15].

Alterations in BP in children have also been associated with OSA, although the evidence is limited. Guilleminault et al. [16] were the first to describe higher BP values among children with OSA. However, Zintzaras et al. [17] published a meta-analysis in 2007 in which they concluded that, until that date, there was insufficient evidence about the relationship between SDB and increased BP. Subsequently, ABPM measurements performed in children with SDB reported elevated systolic and diastolic BP, both during the day and night, independent of obesity status [18,19]. To date, few studies have explored how surgery could improve cardiovascular parameters in children with sleep apnea. A significant decrease was observed in systolic and diastolic BP in children with hypertension and OSA after adeno-tonsillectomy compared to non-hypertensive children [20–25].

From the results provided by these studies, it can be deduced that adeno-tonsillectomy could reduce BP in patients with OSA. However, important limitations in most of them (such as small populations, absence of ABPM in most of them, absence of a control group, etc.) make studies in larger population series and with adequate methodology necessary. For this reason, we set ourselves the main objective of evaluating the BP values present in children with SDB and their response to their treatment.

2. Methodology/Design

This protocol has been registered at NCT03696654 [26]. Hypothesis: sleep-disordered breathing increases the prevalence of arterial hypertension in pediatric patients. This hypertension is reversible after treatment.

2.1. Primary Objective

To demonstrate how the presence of sleep-disordered breathing (SDB) is associated with a higher risk of high blood pressure (HBP) in pediatric patients and to confirm that this is reversible with treatment.

2.2. Secondary Objectives

- Establish the relationship between the presence of HBP and the severity of OSA (apnea–hypopnea index—AHI, Hypoxic load and desaturation index—DI).
- Evaluate the variability along the circadian rhythm of the HBP patterns produced in pediatric patients with SDB.
- Establish the correlation between the diagnosis of HBP measured in the office and by ambulatory control of BP.
- Assess the organic damage produced:
    - Evaluate the manifestation of subclinical organ damage through other markers such as: blood biomarkers (creatinine/glomerular filtration rate), urine (albuminuria/proteinuria), and echocardiography (left ventricular hypertrophy).
    - Establish the pathophysiological mechanisms involved in the HBP/SDB relationship.

2.3. Design and Population

This is a multicenter, longitudinal, prospective study with a control group. A total of 286 children between 4 and 18 years old referred prospectively to undergo a sleep study due to suspected SDB will be included. The study will be directed by the coordinating center (Hospital Universitario de Guadalajara), which will be responsible for the study design and patient follow-up. The other participant centers will be Hospital Universitario Fundación

Jiménez Díaz, Instituto del Sueño, Hospital Universitario Santa Lucía, Hospital San Pedro, and Hospital Universitario de Araba.

2.3.1. Inclusion Criteria

- Approval of the Ethics and Clinical Trials Committee.
- Informed consent signed by parents and/or legal guardians.
- Children between 4 and 18 years old will be evaluated consecutively for suspected SDB.

2.3.2. Exclusion Criteria

- Associated comorbidities: cardiovascular disease (including cardiac malformation), cerebrovascular disease, or unstable severe or exacerbated respiratory disease that preclude the realization of the studies.
- Genetic diseases according to investigator criteria.
- Children with chronic insomnia and/or depressive syndrome.
- Children with malformation syndromes (including craniofacial malformations), Down syndrome, and neuromuscular diseases.
- Previous otorhinolaryngologic surgery and/or CPAP.
- Contraindication for realization of ABPM (arrhythmias, allergy to latex, or coagulation disorders).

Children evaluated for suspected SDB participating in the research study had to meet all the inclusion criteria and none of the exclusion criteria. After informed consent was signed by their parents, the following procedures were developed.

2.4. Procedures

Different clinical and anthropometric variables will have been will be collected, and the diagnosis of SDB will have been established by complete PSG and the diagnosis of HBP by taking BP in the office and 24-h ABPM.

2.4.1. Full Polysomnography

PSG will be performed according to the criteria of the American Academy of Sleep Studies (AASM 2017). Different signals will be recorded, such as nasal flow, snoring, thermistor, thoracic and abdominal movement, transcutaneous capnography, oxygen saturation, heart rate by electrocardiogram, body position, and leg movement. Electroencephalogram recordings will include six electrodes, referred to as contralateral mastoids (A1–A2), adopting the 10–20 rules of international EEG system: two frontal (F3–F4), two central (C3–C4), and two occipital (O1–O2) locations. One ground electrode and another reference

electrode (Cz) will be included. Two chin electrodes will be used to obtain the electromyogram signal and two different electrodes placed above the left and right outer eye cantus will be employed to record the electrooculogram (EOG). Apnea is defined as a flow decrease > 90% in two respiratory cycles for obstructive and >20 s or two respiratory cycles accompanied by a desaturation of 3% in central apnea. Hypopnea is defined as a 90–30% flow decrease in two respiratory cycles accompanied by a desaturation greater than 3% or microarousal (AASM 2017). The AHI is defined as the summatory of apneas and hypopneas divided by the sleep time. Based on the results, four groups will be created based on the severity of the SDB as measured by the AHI: group I: AHI < 3/h; group II: AHI ≥ 3 <5/h; group III: AHI ≥ 5/h <10/h; group IV: AHI ≥ 10/h. Oxygen variables will be calculated automatically. Nocturnal oximetry for oxygen parameters.

2.4.2. Blood Pressure Measurement

Office BP will have been will be measured at the clinic. BP will be measured on three occasions, with a pediatric sphygmomanometer validated for pediatric age using the non-dominant arm, the same day as the ABPM (to be will be used for calibration of the ABPM), and at the post-treatment follow-up visit. The patient will need to be seated for at least 5 min before the BP measures and to remain seated with uncrossed legs and an empty bladder in a quiet environment. Three BP measurements will be taken every 3 min, discarding the first one and averaging the last two.

The ABPM study and the BP data collection will be done following the recommendations of the European Guide for the management of hypertension in children [11]. Hypertension will be considered when systolic blood pressure (SBP) and/or diastolic blood pressure (DBP) are persistently above the 95th percentile according to gender, age, and height and, depending on the distribution of percentiles, the hypertension classification will be done. The ABPM will be conducted a maximum of fifteen days after the PSG study.

Validated pediatric BP monitoring equipment with sleeves appropriate to the size of the child’s arm will be used (the size of the cuff will be calculated by the average distance between the acromion and the radial head). The device is placed on the non-dominant arm and parents will be instructed on how to handle the device (how to turn it off in case of excessive pressure, need to keep the arm still during measurements) and everything related to the test will be explained.

For the study of the circadian rhythm pattern in blood BP, a decrease in SBP and DBP of at least 10% (mean BP during the day − mean BP during the night/mean BP during the day × 100) will be considered normal.

2.5. Visits and Follow-Up

A full sleep study (PSG) will be performed at the sleep unit in the basal visit. The night of the sleep study, parents will be provided with the Chervin questionnaire, answering questions (Yes/No) related to the child’s behavior both during sleep and while awake. They referred

to the habitual behavior of the child in the cardinal or fundamental symptoms of SDB. At the same time, anthropometric measurements will be collected by nurses (V1).

For the HBP study, BP will be taken in the office and 24 h-ABPM (V2).

All patients will be offered to participate voluntarily in the determination of subclinical organ damage related to hypertension and the pathophysiological mechanisms involved, which will be carried out within a maximum period of one month around the performance of each sleep study (and might coincide with the ABPM study).

Once the sleep study is completed, the therapeutic decision will be made in the pediatric clinic based on the criteria established in accordance with the Spanish Respiratory Society-SEPAR sleep-disordered breathing in children consensus (V3) [4].

In order to assess the impact of SDB treatment on BP, measurements will be repeated after therapeutic application (V4). In patients who do not require treatment or are referred for medical or orthodontic treatment, the tests will be repeated 6 months after the therapeutic decision (ABPM, PSG, and organ damage studies, if applicable). In patients referred for adeno-tonsillar surgery, the procedures will be repeated just before the intervention (ABPM) and six months after it (ABPM, PSG, and organ damage studies if applicable). Thus, a control group will be available without impeding the treatment of any patient and without allowing delays in its application linked to the study (Figure 1).

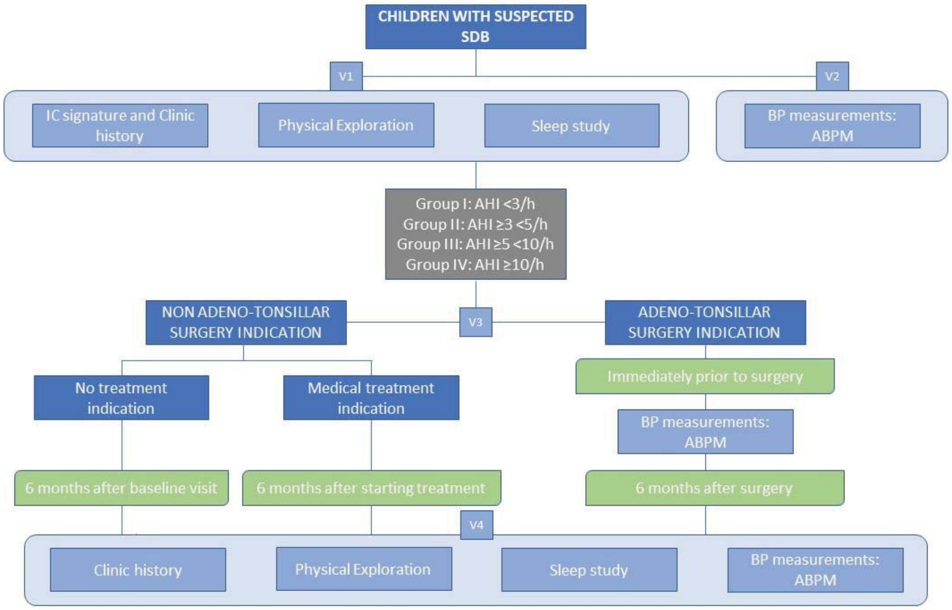

Figure 1. Flow diagram for the study procedure. Abbreviations: SDB, sleep-disordered breathing; IC, informed consent; BP, blood pressure; ABPM, ambulatory blood pressure monitoring; AHI, apnea hypopnea index.

2.6. Study Variables and Data Collection

Data collection will be carried out in a database created for this purpose. Here, the variables that participate in the study will be registered and stored.

The clinical variables will be collected through the Chervin questionnaire, a validate questionnaire usually used in research to identify the presence of SDB in children and to identify important symptoms, including related behavioral disturbances, snoring, and daytime sleepiness [27].

In the physical examination, data of weight, height, body mass index (BMI), neck, hip, and waist circumference, micro-retrognathia (Yes/No), Mallampati (I, II, III, III/IV), tonsillar hypertrophy (I, II, III/IV), adenoid hypertrophy (Yes/No), ogival palate (Yes/No), and bite (III/II/open/asymmetric) will be taken.

BMI will be corrected for weight and height using established guidelines for converting BMI into percentiles. Based on their BMI, children are classified as: underweight (below the 5th percentile); normal weight (between the 5th and 85th percentile); overweight (between the 85th and 95th percentile); or obese (above the 95th percentile). The sleep study variables included will be AHI, DI, minimum saturation, percentage of time below 90% saturation (T90), daytime mean saturation, nighttime mean saturation, sleep efficiency, different sleep states (N1, N2, N3-NREM, and REM), arousal number and index, and leg movement number and index. Central AHI (events with absence of thoracic-abdominal movement) and obstructive AHI (events with presence of thoracic-abdominal movement) will be included. Polysomnographic variables will be collected through PSG performed during the night period. The PSG will be considered valid if it has > 300 valid minutes and >180 min of sleep.

The variability of BP in the circadian rhythm will be analyzed by means of ABPM for 24 h, collecting data on SBP and DBP in the office, mean SBP (SBPm), and mean DBP (DBPm) globally, during the day and night, and the non-dipper pattern.

Ambulatory BP measurements will be taken every 20 min during the day and every 30 min during the night. A study will be considered interpretable when it has at least one reading per hour, 50 readings in 24 h, and 65% of the programmed readings. The test will be repeated if it is not valid.

For the determination of subclinical organ damage, the following determinations will be included: biomarkers (from blood and urine specimens), electrocardiogram, and thoracic echocardiography (left ventricle hypertrophy—LVH data).

The diagnosis of kidney damage due to HBP will be principally made by measuring urine albumin and calculating the glomerular filtration rate (calculated according to blood creatinine, age, and height).

LVH is the most extensively documented marker of organ damage caused by HBP in the pediatric group. Early assessment of LVH in children with HBP is currently recommended as it might facilitate primary prevention of cardiovascular disease. The measure used will be the left ventricular mass index (LVMI) (g/m2), considering LVH when this index is ≥95th

percentile (38.6 g/m2). Between 30–40% of children with HBP will have a LVMI above the 95th percentile and in 10–15% this hypertrophy severe (>51 g/m2).

2.7. Sample Size Calculation

The sample size was calculated by taking the values reported by Ng DK et al. as a reference [24]. For an alpha risk of 0.05 and beta risk of 0.2 in a bilateral contrast for repeated measures, assuming the standard deviation of the variable in the reference group and with an estimated loss to follow-up of 20%, 286 patients were required to detect a decrease of 2 mmHg in SBPm.

The analysis will be carried out with the programs SPSS version 26.0 (IMB SPSS Statistics, CA, USA) and R 2.6.2 (2008, R Foundation for Statistical Computing, Vienna, Austria), accepting a value of $p < 0.05$ as the significance level. The results will be presented as mean ± standard deviation or percentage, according to the type of variable. The adjustment of the quantitative variables to the normal distribution will be evaluated using the Kolmogorov–Smirnof test. For comparisons between groups, the chi-square or t-Student tests will be used.

2.8. Ethical Considerations

This study does not entail relevant risks, except for the discomfort of performing the ABPM study and performing the sleep study control. The ABPM study involves wearing a BP sleeve for 24 h with inflation every 20–30 min. The extraction of biological samples was optional only for those patients who wish to participate in the organ damage sub-study. The study was approved by the Ethics and Clinical Trials Committee (P02/18) and the parents/children signed an informed consent (IC) form. A specific IC was collected for blood and urine samples and biobank storage. Finally, although the study is risk-free, the patients were covered by the general insurance of the National Health System of each participating autonomous community.

3. Relevance of the Study

The management of HBP in pediatric patients continues to be a pending issue in routine clinical practice, even though the presence of high BP levels in children (also values close to normal) has been shown to trigger the progression of hypertension in adults and have a significant association with increased cardiometabolic risk and coronary heart disease in the future.

On the other hand, there is evidence of the involvement of SDB in the presence of HBP levels, relating these SDB with the progression of cardiovascular diseases. Adeno-tonsillectomy in children with AOS has been recently described to significantly reduce the BP, similarly to adult CPAP treatment. However, the treatment in adults has not been proven to reduce cardiovascular events.

If it is shown that BP increases because of SDB, and that this is reversible after treatment, this would have direct implications for the management of SDB in children and could provide fundamental information on the treatment indications for existing clinical guidelines. Thus, it is important to assess the correlation between SDB in children and future cardiovascular risk, as it could have a relevant impact in clinical practice.

Besides, this information could have enormous relevance for the management of sleep apnea in adults. If the hypothesis of our studies is confirmed, it would imply that the treatment for OSA in children would have a beneficial effect on reducing their future heart attack risk.

Children, unlike adults, are an optimal population because they have no associated risk factors that could act as confounding factors (naïve condition), they have an effective treatment, and they allow for the natural history of the disease to be studied. As a result, they constitute an ideal target for this research, which will also be useful in the management of adults.

For all the above, we advocate that the KIDS TRIAL study constitutes a clear translational study with high potential clinical applicability value.

References

1. Marcus, C.L.; Brooks, L.J.; Ward, S.D.; Draper, K.A.; Gozal, D.; Halbower, A.C.; Jones, J.; Lehmann, C.; Schechter, M.S.; Sheldon, S.; et al. Diagnosis and Management of Childhood Obstructive Sleep Apnea Syndrome. *Pediatrics* **2012**, *130*, e714–e755. https://doi.org/10.1542/peds.2012-1672.

2. American Thoracic Society. Standards and Indications for Cardiopulmonary Sleep Studies in Children. American Thoracic Society. *Am. J. Respir. Crit. Care Med.* **1996**, *153*, 866–878. https://doi.org/10.1164/ajrccm.153.2.8564147.

3. Blechner, M.; Williamson, A.A. Consequences of Obstructive Sleep Apnea in Children. *Curr. Probl. Pediatr. Adolesc. Health Care* **2016**, *46*, 19–26. https://doi.org/10.1016/j.cppeds.2015.10.007.

4. Luz Alonso-Álvarez, M.; Canet, T.; Cubell-Alarco, M.; Estivill, E.; Fernández-Julián, E.; Gozal, D.; Jurado-Luque, M.J.; Lluch-Roselló, M.A.; Martínez-Pérez, F.; Merino-Andreu, M.; et al. Documento de Consenso Del Síndrome de Apneas-Hipopneas Durante El Sueño En Niños (Versión Completa). *Arch. Bronconeumol.* **2011**, *47*, 2–18. https://doi.org/10.1016/S0300-2896(11)70026-6.

5. Lumeng, J.C.; Chervin, R.D. Epidemiology of Pediatric Obstructive Sleep Apnea. *Proc. Am. Thorac. Soc.* **2008**, *5*, 242–252. https://doi.org/10.1513/pats.200708-135MG.

6. Marin JM, Carrizo SJ, Vicente E, Agusti AG. Long-term cardiovascular outcomes in men with obstructive sleep apnoea-hypopnoea with or without treatment with continuous positive airway pressure: an observational study. Lancet. **2005**;365(9464):1046-1053. doi:10.1016/S0140-6736(05)71141-7. doi: 10.1016/S0140-6736(05)71141-7

7. Martínez-García, M.-A.; Capote, F.; Campos-Rodríguez, F.; Lloberes, P.; Díaz de Atauri, M.J.; Somoza, M.; Masa, J.F.; González, M.; Sacristán, L.; Barbé, F.; et al. Effect of CPAP on Blood Pressure in Patients With Obstructive Sleep Apnea and Resistant Hypertension. *JAMA* **2013**, *310*, 2407. https://doi.org/10.1001/jama.2013.281250.

8. Lorenzi Filho, G.; Genta, P.R.; Pedrosa, R.P.; Drager, L.F.; Martinez, D. Consequências Cardiovasculares Na SAOS. *J. Bras. Pneumol.* **2010**, *36* (Suppl. 2), 38–42. https://doi.org/10.1590/S1806-37132010001400011.

9. Barbé, F.; Durán-Cantolla, J.; Sánchez-de-la-Torre, M.; Martínez-Alonso, M.; Carmona, C.; Barceló, A.; Chiner, E.; Masa, J.F.; Gonzalez, M.; Marín, J.M.; et al. Effect of Continuous Positive Airway Pressure on the Incidence of Hypertension and Cardiovascular Events in Nonsleepy Patients With Obstructive Sleep Apnea: A Randomized Controlled Trial. *JAMA* **2012**, *307*, 2161–2168. https://doi.org/10.1001/jama.2012.4366.

10. McEvoy, R.D.; Antic, N.A.; Heeley, E.; Luo, Y.; Ou, Q.; Zhang, X.; Mediano, O.; Chen, R.; Drager, L.F.; Liu, Z.; et al. CPAP for Prevention of Cardiovascular Events in Obstructive Sleep Apnea. *N. Engl. J. Med.* **2016**, *375*, 919–931. https://doi.org/10.1056/NEJMoa1606599.

11. Lurbe, E.; Agabiti-Rosei, E.; Cruickshank, J.K.; Dominiczak, A.; Erdine, S.; Hirth, A.; Invitti, C.; Litwin, M.; Mancia, G.; Pall, D.; et al. 2016 European Society of Hypertension Guidelines for the Management of High Blood Pressure in Children and Adolescents. *J. Hypertens.* **2016**, *34*, 1887–1920. https://doi.org/10.1097/HJH.0000000000001039.

12. National High Blood Pressure Education Program Working Group on High Blood Pressure in Children and Adolescents. The Fourth Report on the Diagnosis, Evaluation, and Treatment of High Blood Pressure in Children and Adolescents. *Pediatrics* **2004**, *114*, 555–576.

13. Tirosh, A.; Afek, A.; Rudich, A.; Percik, R.; Gordon, B.; Ayalon, N.; Derazne, E.; Tzur, D.; Gershnabel, D.; Grossman, E.; et al. Progression of Normotensive Adolescents to Hypertensive Adults. *Hypertension* **2010**, *56*, 203–209. https://doi.org/10.1161/HYPERTENSIONAHA.109.146415.

14. Campana, E.M.G.; Brandão, A.A.; Pozzan, R.; França, M.D.F.; Fonseca, F.L.; Pizzi, O.L.; Magalhães, M.E.C.; de Freitas, E.V.; Brandão, A.P. Pressão Arterial Em Jovens Como Marcador de Risco Cardiovascular. Estudo Do Rio de Janeiro. *Arq. Bras. Cardiol.* **2009**, *93*, 657–665. https://doi.org/10.1590/S0066-782X2009001200016.

15. Erlingsdottir, A.; Indridason, O.S.; Thorvaldsson, O.; Edvardsson, V.O. Blood Pressure in Children and Target-Organ Damage Later in Life. *Pediatr. Nephrol.* **2010**, *25*, 323–328. https://doi.org/10.1007/s00467-009-1350-3.

16. Guilleminault, C.; Eldridge, F.L.; Simmons, F.B.; Dement, W.C. Sleep Apnea in Eight Children. *Pediatrics* **1976**, *58*, 23–30.

17. Zintzaras, E.; Kaditis, A.G. Sleep-Disordered Breathing and Blood Pressure in Children. *Arch. Pediatr. Adolesc. Med.* **2007**, *161*, 172. https://doi.org/10.1001/archpedi.161.2.172.

18. Li, A.M.; Au, C.T.; Sung, R.Y.T.; Ho, C.; Ng, P.C.; Fok, T.F.; Wing, Y.K. Ambulatory Blood Pressure in Children with Obstructive Sleep Apnoea: A Community Based Study. *Thorax* **2008**, *63*, 803–809. https://doi.org/10.1136/thx.2007.091132.

19. Kang, K.-T.; Chiu, S.-N.; Weng, W.-C.; Lee, P.-L.; Hsu, W.-C. Analysis of 24-Hour Ambulatory Blood Pressure Monitoring in Children With Obstructive Sleep Apnea. *Medicine* **2015**, *94*, e1568. https://doi.org/10.1097/MD.0000000000001568.

20. Amin, R.; Anthony, L.; Somers, V.; Fenchel, M.; McConnell, K.; Jefferies, J.; Willging, P.; Kalra, M.; Daniels, S. Growth Velocity Predicts Recurrence of Sleep-Disordered Breathing 1 Year after Adenotonsillectomy. *Am. J. Respir. Crit. Care Med.* **2008**, *177*, 654–659. https://doi.org/10.1164/rccm.200710-1610OC.

21. Kuo, Y.-L.; Kang, K.-T.; Chiu, S.-N.; Weng, W.-C.; Lee, P.-L.; Hsu, W.-C. Blood Pressure after Surgery among Obese and Nonobese Children with Obstructive Sleep Apnea. *Otolaryngol.–Head Neck Surg.* **2015**, *152*, 931–940. https://doi.org/10.1177/0194599815573927.

22. Lee, L.-A.; Li, H.-Y.; Lin, Y.-S.; Fang, T.-J.; Huang, Y.-S.; Hsu, J.-F.; Wu, C.-M.; Huang, C.-G. Severity of Childhood Obstructive Sleep Apnea and Hypertension Improved after Adenotonsillectomy. *Otolaryngol.–Head Neck Surg.* **2015**, *152*, 553–560. https://doi.org/10.1177/0194599814561203.

23. Ng, D.K.; Wong, J.C.; Chan, C.; Leung, L.C.K.; Leung, S. Ambulatory Blood Pressure before and after Adenotonsillectomy in Children with Obstructive Sleep Apnea. *Sleep Med.* **2010**, *11*, 721–725. https://doi.org/10.1016/j.sleep.2009.10.007.

24. Apostolidou, M.T.; Alexopoulos, E.I.; Damani, E.; Liakos, N.; Chaidas, K.; Boultadakis, E.; Apostolidis, T.; Gourgoulianis, K.; Kaditis, A.G. Absence of Blood Pressure, Metabolic, and Inflammatory Marker Changes after Adenotonsillectomy for Sleep Apnea in Greek Children. *Pediatr. Pulmonol.* **2008**, *43*, 550–560. https://doi.org/10.1002/ppul.20808.

25. Crisalli, J.A.; McConnell, K.; VanDyke, R.D.; Fenchel, M.C.; Somers, V.K.; Shamszumann, A.; Chini, B.; Daniels, S.R.; Amin, R.S. Baroreflex Sensitivity after Adenotonsillectomy in Children with Obstructive Sleep Apnea during Wakefulness and Sleep. *Sleep* **2012**, *35*, 1335–1343. https://doi.org/10.5665/sleep.2108.

26. ClinicalTrial.gov. Available online: https://clinicaltrials.gov/ct2/show/record/NCT03696654 (accessed on 28 November 2017).

27. Chervin, R.D.; Hedger, K.; Dillon, J.E.; Pituch, K.J. Pediatric Sleep Questionnaire (PSQ): Validity and Reliability of Scales for Sleep-Disordered Breathing, Snoring, Sleepiness, and Behavioral Problems. *Sleep Med.* **2000**, *1*, 21–32. https://doi.org/10.1016/S1389-9457(99)00009-X.

1 Study hypothesis

Sleep-disordered breathing (SDB) increases the prevalence of arterial hypertension (AHT) in pediatric patients. Such hypertension is reversible after treatment.

2 Objectives

2.1 Main objective

The main objective is to **evaluate the association of the presence of sleep-disordered breathing with the risk of hypertension** in pediatric patients, as well as to **verify that hypertension is reversible with treatment**.

2.2 Secondary objectives

The secondary objectives are as follows:

1. **To estimate the relationship between the presence of ETS and the severity of sleep apnea-hypopnea syndrome (SAHS)**, assessed by the apnea-hypopnea index (AHI) and the desaturation index.
2. **To study the variability along the circadian rhythm of ETS patterns** produced in pediatric patients with sleep-disordered breathing disorders.
3. **To estimate the association between the diagnosis of hypertension measured in consultation and by ambulatory blood pressure control**.
4. To **describe subclinical organ damage** by means of other renal and cardiovascular markers (creatinine, glomerular filtration rate, albuminuria, proteinuria, left ventricular hypertrophy) and to **establish the pathophysiological mechanisms involved in the AHT-TRS relationship.**
5. **Validate pulse transit time (PTT) measurement as blood pressure monitoring**.

2.3 Objectives addressed in this report

As indicated by the research team, this report presents the results of the following specific objectives:

1. **To describe** the sociodemographic and clinical variables recorded at the baseline visit of the study population, as well as the results of polysomnography, ambulatory blood pressure monitoring (ABPM) and the organic damage study.
2. **Describe** the therapeutic decision made based on previous test results.
3. **To evaluate** the association between the severity of sleep apnea-hypopnea syndrome (SAHS) and blood pressure measured with ABPM, and to **estimate** its impact on the presence of *non-dipper* pattern and the lowering of mean arterial pressure (MAP).
4. **To evaluate** the association between SAHS severity and left ventricular hypertrophy.
5. **To evaluate** the association between SAHS severity and organ damage.

6. **To evaluate** the association between the severity of arterial hypertension (AHT) and the hypoxic load measured by the number and index of desaturations.
7. **To estimate** the correlation between blood pressure measurements in consultation with those obtained in the ABPM.
8. In the population with AHI≥3/h, **compare** clinical characteristics (Chervin questionnaire), physical examination, polysomnography, ABPM and echocardiogram data, and left ventricular hypertrophy between boys and girls.
9. **To evaluate** the association between the severity of hypoxic TST load and blood pressure measured with MAPA, and to **estimate** its impact on the presence of *non-dipper* pattern and lowering of MAP.
10. **To evaluate** the association between the severity of hypoxic TST load and left ventricular hypertrophy.
11. **To evaluate** the association between the severity of hypoxic TST load and organ damage.
12. **To evaluate** the association between the severity of REM hypoxic load and blood pressure measured with MAPA, and to **estimate** its impact on the presence of *non-dipper* pattern and MAP lowering.
13. **To evaluate** the association between severity of REM hypoxic load and left ventricular hypertrophy.
14. **To evaluate** the association between severity of REM hypoxic load and organ damage.
15. **To evaluate** the association between the severity of non-REM hypoxic load and blood pressure measured with MAPA, and to **estimate** its impact on the presence of *non-dipper* pattern and MAP lowering.
16. **To evaluate** the association between severity of non-REM hypoxic load and left ventricular hypertrophy.
17. **To evaluate** the association between the severity of non-REM hypoxic load and organ damage.
18. **To evaluate** the association between the severity of supine hypoxic load and blood pressure measured with MAPA, and to **estimate** its impact on the presence of *non-dipper* pattern and lowering of MAP.
19. **To evaluate** the association between severity of supine hypoxic load and left ventricular hypertrophy.
20. **To evaluate** the association between severity of supine hypoxic load and organ damage.
21. **To evaluate** the association between the severity of non-supine hypoxic load and blood pressure measured with MAPA, and to **estimate** its impact on the presence of *non-dipper* pattern and lowering of MAP.
22. **To evaluate** the association between severity of nonsupine hypoxic loading and left ventricular hypertrophy.
23. **To evaluate** the association between severity of non-supine hypoxic load and organ damage.
24. **To evaluate** the discriminative capacity of the hypoxic load for the presence of the *non-dipper* pattern.

3 Study design

Observational, prospective, multicenter, national, prospective study. The study was conducted under routine clinical practice conditions.

4 Patient selection criteria

Patients were selected based on the following criteria.

Inclusion criteria:

1. Children aged 4 to 18 years evaluated for suspected SRT.
2. Patients whose parents and/or guardians have signed the informed consent to participate in the study.

Exclusion criteria:

1. Patients with any of the following associated comorbidities: cardiovascular disease (including cardiac malformation, unstable or exacerbated severe cerebrovascular or respiratory disease that makes it impossible to perform the study tests.
2. Patients with genetic diseases.
3. Patients with chronic insomnia and/or depressive syndrome.
4. Patients with malformations (including craniofacial malformations), Down syndrome, or neuromuscular diseases.
5. Patients with a history of otorhinolaryngology and/or previous CPAP.
6. Patients with any type of contraindication for MAPA/PTT (arrhythmias, latex allergy or coagulation disorders).

5 Patients included in the study and assessable

The table of exclusions is shown below. Of the 190 patients included in the registry **with measurements of hypoxic load (TST)**, 190 were evaluable.

Table 1: Exclusions and patients to be evaluated

		n
Patients included		190
Exclusions	*Do not meet inclusion criterion #1*	0
	Do not meet inclusion criterion #2	0
	Meet exclusion criterion #1	0
	Meet exclusion criterion #2	0
	Meet exclusion criterion #3	0
	Meet exclusion criterion #4	0
	Meet exclusion criterion #5	0
	Meet exclusion criterion #6	0
**Patients to be assessed**		**190**

6 Calculation and recoding of variables

Some variables have been calculated from data collected in the electronic CRD.

Age at inclusion: Quantitative variable, in years, obtained as the years elapsed between the date of birth and the date of signing the informed consent.

Mean SAD: Quantitative variable, in mmHg, obtained as the arithmetic mean of the second and third SAD measurements in consultation.

Mean DBT: Quantitative variable, in mmHg, obtained as the arithmetic mean of the second and third measurement of DBT in consultation.

***Chervin* score**: Dichotomous variable obtained based on the responses to the *Chervin* questionnaire. Responses are scored as follows:

i. In blocks A and B: "Yes" adds up to 1; "No" adds up to 0; "NS" is not included in the total number of questions answered (the same as if it had not been answered);
ii. In block C: "Many times" or "Almost always" adds up to 1; "Never" or "Sometimes" adds up to 0; unanswered questions are not included in the total number of questions answered;

The total score is divided by the number of questions answered (and with an answer other than "NS"). The score obtained is dichotomized, so that:

Positive (abnormal) ⇔ division is > 0.33.
Negative (normal) ⇔ the division is ≤ 0.33.

SAHS risk groups (*recalculated due to errors in the BdD categorization*): Qualitative variable obtained based on AHI /h measurements, such that:

Group I: no TRS ⇔ IAH /h <3
Group II: AHI 3-5/h ⇔ 3 ≤ AHI /h <5
Group III: AHI>5/h ⇔ 5 ≤ AHI /h <10
Group IV: AHI>10/h ⇔ AHI /h ≥ 10

PAM drop (*recalculated due to errors in the observations of the BoD*): Quantitative variable, in %, obtained as:

$$100 \times \frac{\mathrm{PAMm_{diurna}} - \mathrm{PAMm_{nocturna}}}{\mathrm{PAMm_{diurna}}}$$

***Non-dipper* pattern** (*recalculated due to errors in the BdD categorization*): Dichotomous variable obtained based on the lowering of the PAM, so that:

Yes ⇔ MAP drop < 10%.
No ⇔ PAM drop ≥ 10%.

Quartiles of baseline hypoxic TST load: Categorical variable based on empirical quartiles of hypoxic TST load at baseline visit.

Quartiles of baseline REM hypoxic load: Categorical variable based on empirical quartiles of REM hypoxic load at baseline visit.

Quartiles of baseline NREM hypoxic load: Categorical variable based on empirical quartiles of NREM hypoxic load at baseline visit.

Quartiles of baseline supine hypoxic load: Categorical variable based on empirical quartiles of supine hypoxic load at baseline visit.

Quartiles of baseline nonsupine hypoxic load: Categorical variable based on empirical quartiles of nonsupine hypoxic load at baseline visit.

7 Sample size

For details of the calculation, see the study protocol.

8 Quality and control of recorded data

The variables necessary to address the study objectives have been recorded by the investigators through an electronic CRD designed specifically for the study. All CRDs were identified by the investigator's code and the patient's code.

The data recorded in the study's electronic CRD have been stored in a database and reviewed for possible inconsistencies and/or missing data to ensure correct completion of the data and thus ensure optimal quality prior to performing the present analysis.

9 Statistical methodology

9.1 Sample analysis

The sample of assessable patients includes all those who meet the selection criteria.

9.2 Handling missing data

No missing *data* imputation has been performed, which causes the loss of cases in analyses involving variables with missing data.

9.3 Analysis by objectives

The analysis of objectives #1 and #2 is descriptive. Frequencies and percentages over the valid N have been obtained for categorical variables and measures of central tendency (mean or median) and dispersion (standard deviation [SD] or 25% [P25] and 75% [P75] percentiles) for continuous variables, according to whether or not they present a normal distribution (evaluated with the

Shapiro-Wilk test). Valid N has been reported for all variables. The results for objective #1 are shown in Tables 2-36, and those for objective #2 in Table 38.

To evaluate the association of SAHS severity with blood pressure measured with ABPM (objective #3), with left ventricular hypertrophy (objective #4) and with organ damage (objective #5), the study variables have been described according to SAHS risk groups and trend tests have been performed considering the ordinality of SAHS risk groups. To estimate the impact of SAHS on the presence of *non-dipper* pattern, a multivariable logistic regression model has been fitted, including sex, age and body mass index (BMI) as adjustment variables. The results are presented as *odds ratios* (OR) together with the 95% confidence interval (CI). Additionally, a multivariable linear regression model has been fitted to estimate the impact of SAHS on the lowering of MAP, in which sex, age and BMI have also been included as adjustment variables. The results of objective #3 are shown in the *SAHS and MAP* section, those of objective #4 in the *SAHS and ultrasound variables* section and those of objective #5 in the *SAHS and organic damage* section.

To evaluate the association between the severity of HT and hypoxic load (objective #6), we proceeded in a manner analogous to the previous objectives. The results are shown in the *ETS and desaturations* section. Finally, we estimated the Spearman correlation together with the 95% CI between the blood pressure measurements in consultation with those obtained in the ABPM (objective #7), which are shown in the section *Blood pressure in consultation and ABPM*.

Comparison by sex of clinical characteristics, physical examination, polysomnography data, ABPM and echocardiogram data, and left ventricular hypertrophy (objective #8) was performed by means of the χ^2 test for categorical variables, and the t test or Mann-Whitney U test for continuous variables, depending on whether or not they present a normal distribution. The results are shown in the section *Comparisons by sex*.

The analysis of objectives #9, #10 and #11 is analogous to that of objectives #3, #4 and #5, but by quartiles of hypoxic load, instead of SAHS risk groups. The results of objective #9 are shown in the section *Hypoxic TST load and MAPA*, those of objective #10 in the section *Hypoxic TST load and ultrasound variables*, and those of objective #11 in the section *Hypoxic TST load and organ damage*.

Additionally, REM hypoxic load (targets #12, #13 and #14), non-REM (targets #15, #16 and #17), supine (targets #18, #19 and #20) and non-supine (targets #21, #22 and #23) have been analyzed; the results are shown in the sections *REM hypoxic load and MAP*, *REM hypoxic load and ultrasound variables*, *REM hypoxic load and organ damage*, *Non-REM hypoxic load and MAP*, *Non-REM hypoxic load and ultrasound variables*, *Non-REM hypoxic load and organ damage*, *Supine hypoxic load and ABPM*, *Supine hypoxic load and ultrasound variables*, *Supine hypoxic load and organ damage*, *Non-supine hypoxic load and ABPM*, *Non-supine hypoxic load and ultrasound variables*, and *Non-supine hypoxic load and organ damage*, respectively. In the subsequent sections, the results are shown by the median of the hypoxic load measures.

The discriminative capacity of the hypoxic load for the presence of the *non-dipper* pattern (specific objective #24) was evaluated by calculating the area under the ROC curve (AUC) and its 95% CI. The cut-off point that maximizes specificity with a sensitivity of 0.8 has been obtained. The results can be found in the section Analysis of the predictive ability of the hypoxic load on the *non-dipper* pattern.
