# Supplementary material for: Cardiovascular Risk Through Hypoxic Burden in Children With Sleep Apnea: A Secondary Analysis of a Nonrandomized Clinical Trial
Source: JAMA Netw Open. 2025 Oct 23;8(10):e2538744. doi: 10.1001/jamanetworkopen.2025.38744 (PMC12550637; doi:10.1001/jamanetworkopen.2025.38744)
Supplement: Supplement 2. — eTable 1. Comparison Between the Excluded Individuals and Those Included in the Analysis eTable 2. Polysomnographic Variables eTable 3. Measures of the Ambulatory 24-Hour Blood Pressure Parameters According to Quartile-Based Groups of HB eTable 4. Measures of the Ambulatory 24-Hour Blood Pressure Parameters According to the Hypoxic Burden in Rapid Eye Movement Sleep eTable 5. Measures of the Ambulatory 24-Hour Blood Pressure Parameters According to the Hypoxic Burden in Non–Rapid Eye Movement Sleep eTable 6. Measures of the Ambulatory 24-Hour Blood Pressure Parameters According to the Hypoxic Burden in the Supine Position eTable 7. Measures of the Ambulatory 24-Hour Blood Pressure Parameters According to the Hypoxic Burden in the Nonsupine Position eFigure 1. Forest plot Showing the Result of the Multivariable Logistic Regression Analysis for Nondipper Pattern According to HB in REM Sleep eFigure 2. Forest plot Showing the Result of the Multivariable Logistic Regression Analysis for Nondipper Pattern According to HB in non-REM Sleep eFigure 3. Forest Plot Showing the Result of the Multivariable Logistic Regression Analysis for Nondipper Pattern According to HB in Supine Position eFigure 4. Forest Plot Showing the Result of the Multivariable Logistic Regression Analysis for Nondipper Pattern According to HB in Nonsupine Position [file jamanetwopen-e2538744-s002.pdf]

Supplementary Online Content

Mediano O, López-Monzoni S, Castillo-García M, et al. Cardiovascular risk through hypoxic burden in children with sleep apnea: a secondary analysis of a nonrandomized clinical trial. *JAMA Netw Open.* 2025;8(10):e2538744. doi:10.1001/jamanetworkopen.2025.38744

eTable 1. Comparison Between the Excluded Individuals and Those Included in the Analysis

eTable 2. Polysomnographic Variables

eTable 3. Measures of the Ambulatory 24-Hour Blood Pressure Parameters According to Quartile-Based Groups of HB

eTable 4. Measures of the Ambulatory 24-Hour Blood Pressure Parameters According to the Hypoxic Burden in Rapid Eye Movement Sleep

eTable 5. Measures of the Ambulatory 24-Hour Blood Pressure Parameters According to the Hypoxic Burden in Non–Rapid Eye Movement Sleep

eTable 6. Measures of the Ambulatory 24-Hour Blood Pressure Parameters According to the Hypoxic Burden in the Supine Position

eTable 7. Measures of the Ambulatory 24-Hour Blood Pressure Parameters According to the Hypoxic Burden in the Nonsupine Position

eFigure 1. Forest plot Showing the Result of the Multivariable Logistic Regression Analysis for Nondipper Pattern According to HB in REM Sleep

eFigure 2. Forest plot Showing the Result of the Multivariable Logistic Regression Analysis for Nondipper Pattern According to HB in non-REM Sleep

eFigure 3. Forest Plot Showing the Result of the Multivariable Logistic Regression Analysis for Nondipper Pattern According to HB in Supine Position

eFigure 4. Forest Plot Showing the Result of the Multivariable Logistic Regression Analysis for Nondipper Pattern According to HB in Nonsupine Position

This supplementary material has been provided by the authors to give readers additional information about their work.

© 2025 Mediano O et al. *JAMA Network Open*.

eTable 1. Comparison Between the Excluded Individuals and Those Included in the Analysis

	Overall	Excluded	Included	*P* for trend
	n=286	n=96	n=190	
**Sex, women, No (%):**	118 (41.3)	36 (37.5)	82 (43.2)	.43
**Age, median (IQR), years**	6.00 (5.00,8.00)	6.00 (5.00,9.00)	6.00 (5.00,7.75)	.04
**BMI percentile, median (IQR)**	49.0 (27.0,86.0)	66.0 (31.0,95.0)	43.5 (24.2,77.8)	.005
**SBP, median (IQR), mmHg**	102 (94.5,109)	102 (96.0,110)	100 (94.5,109)	.28
**DBP, median (IQR), mmHg**	65.5 (60.0,70.5)	63.0 (60.0,68.5)	66.2 (60.5,71.5)	.02
**PSQ positive (abnormal), No (%)**	176 (62.0)	64 (68.1)	112 (58.9)	.17
**TST, median (IQR), min**	430 (400,465)	422 (394,476)	431 (402,463)	.72
**Sleep efficiency, median (IQR), % TST**	91.0 (85.3,95.5)	92.5 (87.2,96.0)	90.9 (84.4,94.9)	.09
**NREM latency, median (IQR), min**	9.80 (3.27,19.7)	11.1 (6.62,26.0)	8.60 (2.00,18.0)	.01
**N1, median (IQR), % TST**	2.40 (0.40,5.60)	1.70 (0.10,4.70)	2.85 (0.62,5.90)	.02
**N2, median (IQR), % TST**	32.6 (19.3,41.5)	33.5 (16.6,43.8)	32.5 (21.5,40.5)	.58
**N3, median (IQR), % TST**	44.0 (33.7,54.6)	44.4 (30.8,58.8)	43.9 (34.8,53.8)	.86
**REM, median (IQR), % TST**	21.2 (16.6,25.2)	20.7 (16.1,25.3)	21.6 (16.7,25.0)	.56
**AHI, median (IQR), events/h**	6.05 (3.20,10.6)	6.55 (3.32,11.1)	5.95 (3.10,10.3)	.61
**Obstructive AHI, No (%), events/h**				.82
**<1**	29 (10.7)	9 (10.7)	20 (10.7)	
**[1, 5)**	109 (40.2)	31 (36.9)	78 (41.7)	
**[5, 10)**	74 (27.3)	26 (31.0)	48 (25.7)	
**≥10**	59 (21.8)	18 (21.4)	41 (21.9)	
**Minimum SaO_2, median (IQR), %**	89.0 (86.0,91.0)	89.5 (86.0,92.0)	89.0 (86.0,91.0)	.49
**Mean SaO_2, median (IQR), %**	96.0 (95.0,97.0)	97.0 (96.0,97.0)	96.0 (95.0,97.0)	<.001

Frequencies and percentages have been obtained for categorical variables and median (IQR) for continuous variables. AHI: apnea-hypopnea index; DBP: diastolic blood pressure; IQR: interquartile range; NREM: non-rapid eye movement; PaCO2: partial pressure of carbon dioxide; PLM: periodic leg movement; REM: rapid eye movement; SaO2: oxygen saturation; SBP: systolic blood pressure; TST: Total sleep time; WASO: wake after sleep onset.

© 2025 Mediano O et al. *JAMA Network Open*.

eTable 2. Polysomnographic Variables

	Overall	Quartile 1: ≤3.82	Quartile 2: (3.82; 9.6]	Quartile 3: (9.6; 22.53]	Quartile 4: >22.53	*P* for trend
	n=190	n=48	n=48	n=46	n=48	
**TST, median (IQR), min**	431 (402,463)	432 (399,453)	431 (402,463)	429 (406,470)	433 (407,460)	.61
**Sleep efficiency, median (IQR), % TST**	90.9 (84.4,94.9)	91.0 (81.0,94.5)	89.0 (83.7,95.0)	91.2 (85.1,95.6)	90.9 (85.1,94.8)	.31
**NREM latency, median (IQR), min**	8.60 (2.00,18.0)	8.45 (2.67,18.0)	9.10 (3.50,19.6)	8.30 (2.00,23.3)	6.50 (0.30,13.4)	.35
**REM latency, median (IQR), min**	112 (73.0,152)	91.0 (72.5,150)	102 (67.1,151)	113 (76.2,170)	122 (75.0,141)	.32
**WASO, median (IQR), min**	31.9 (15.7,58.5)	34.5 (17.5,66.0)	35.1 (17.4,62.8)	27.0 (17.0,56.5)	30.6 (12.8,55.5)	.30
**N1, median (IQR), % TST**	2.85 (0.62,5.90)	2.95 (0.65,6.35)	2.55 (0.58,5.30)	3.80 (1.05,5.92)	1.85 (0.40,5.90)	.97
**N2, median (IQR), % TST**	32.5 (21.5,40.5)	32.5 (22.1,43.9)	32.4 (19.3,41.3)	33.1 (23.5,39.6)	32.4 (21.4,37.8)	.57
**N3, median (IQR), % TST**	43.9 (34.8,53.8)	44.1 (34.5,54.2)	40.2 (33.6,54.4)	44.1 (35.8,52.5)	44.6 (35.2,51.5)	.84
**REM, median (IQR), % TST**	21.6 (16.7,25.0)	19.9 (16.1,25.2)	22.7 (16.9,25.3)	21.2 (17.2,25.1)	20.8 (17.0,23.9)	.98
**Total arousal index, median (IQR)**	11.9 (9.00,17.4)	10.3 (7.65,13.2)	11.4 (9.30,15.7)	14.1 (10.9,19.0)	14.2 (9.50,22.0)	<0.001
**Respiratory arousal index, median (IQR)**	1.80 (0.80,3.65)	0.80 (0.27,1.88)	1.50 (0.70,2.47)	2.00 (1.10,4.10)	3.10 (1.98,6.65)	<0.001
**Spontaneous arousal index, median (IQR)**	4.90 (3.40,7.60)	4.45 (3.25,7.32)	4.95 (3.55,7.67)	6.00 (4.40,9.05)	4.95 (3.18,7.28)	.37
**Arousal index by PLM, median (IQR)**	3.90 (2.50,5.15)	3.95 (2.55,4.82)	4.05 (2.77,5.40)	3.90 (1.95,5.60)	3.70 (2.38,5.35)	.68
**AHI, median (IQR), events/h**	6.0 (3.1,10.3)	2.6 (1.4, 4.7)	4.7 (3.0,7.1)	7.0 (4.6, 10.4)	13.3 (9.9, 25.6)	<0.001
**Supine AHI, median (IQR), events/h**	6.1 (3.1, 11.7)	3.0 (1.5,6.3)	4.8 (3.0,7.7)	7.3 (5.4, 12.1)	18.3 (7.5, 32.3)	<0.001
**Non-supine AHI, median (IQR), events/h**	5.1 (2.4, 9.8)	2.1 (0.9, 3.4)	3.8 (2.4, 6.0)	5.4 (3.5, 9.2)	14.0 (9.5, 21.7)	<0.001

© 2025 Mediano O et al. *JAMA Network Open*.

	Overall	Quartile 1: ≤3.82	Quartile 2: (3.82; 9.6]	Quartile 3: (9.6; 22.53]	Quartile 4: >22.53	*P* for trend
	n=190	n=48	n=48	n=46	n=48	
**Central AHI, median (IQR), events/h**	0.9 (0.4, 1.6)	0.5 (0.3, 0.8)	1.0 (0.5, 1.8)	0.9 (0.5, 1.6)	1.1 (0.6,1.8)	<0.001
**Obstructive AHI, median (IQR), events/h**	4.7 (2.2, 9.2)	1.9 (0.9, 4.3)	3.1 (1.9, 5.4)	5.6 (3.1, 9.8)	11.9 (7.9, 24.4)	<0.001
**Obstructive AHI classification, No (%) events/h**						
**<1**	20 (10.7)	13 (27.7)	6 (12.8)	1 (2.17)	0 (0)	
**[1, 5)**	78 (41.7)	27 (57.4)	26 (55.3)	19 (41.3)	6 (12.8)	
**[5, 10)**	48 (25.7)	7 (14.9)	14 (29.8)	15 (32.6)	12 (25.5)	
**≥10**	41 (21.9)	0 (0)	1 (2.13)	11 (23.9)	29 (61.7)	
**Baseline $PaCO_2$, median (IQR), mmHg**	36.1 (33.9,39.0)	35.3 (33.5,37.7)	35.8 (33.2,39.1)	37.2 (34.4,39.0)	36.6 (34.0,39.7)	.26
**Maximum $PaCO_2$, median (IQR), mmHg**	45.0 (41.0,49.0)	44.0 (40.0,47.0)	45.0 (43.0,48.0)	45.0 (43.0,49.0)	46.0 (41.0,50.8)	.14
**$PaCO_2$ > 50 mmHg, , median (IQR), %**	0.00 (0.00,0.00)	0.00 (0.00,0.00)	0.00 (0.00,0.00)	0.00 (0.00,0.00)	0.00 (0.00,5.88)	.01
**Minimum SaO_2, median (IQR), %**	89.0 (86.0,91.0)	92.0 (89.8,92.0)	90.0 (88.0,91.0)	88.5 (86.0,90.0)	85.5 (80.8,89.0)	<.001
**Mean SaO_2, median (IQR), %**	96.0 (95.0,97.0)	96.0 (95.0,97.0)	96.0 (95.8,97.0)	95.0 (95.0,96.0)	95.0 (94.0,96.0)	<.001
**Desaturation index, , median (IQR)**	5.30 (3.20,10.5)	2.50 (1.15,3.80)	4.30 (2.98,5.60)	6.60 (4.80,12.2)	13.1 (10.1,20.3)	<.001

Frequencies and percentages have been obtained for the categorical variables and median (IQR) for the continuous variables. AHI: apnea-hypopnea index; HB: hypoxic burden; IQR: interquartile range; NREM: non-rapid eye movement; PaCO2: partial pressure of carbon dioxide; PLM: periodic leg movement; REM: rapid eye movement; SaO2: oxygen saturation; TST: Total sleep time; WASO: wake after sleep onset.

© 2025 Mediano O et al. *JAMA Network Open*.

eTable 3. Measures of the Ambulatory 24-Hour Blood Pressure Parameters According to Quartile-Based Groups of HB

ABPM parameters	Overall	HB groups (based in quartiles)				*P* for trend
		Quartile 1: ≤3.82	Quartile 2: (3.82; 9.6]	Quartile 3: (9.6; 22.53]	Quartile 4: >22.53	
	n=187	n=47	n=47	n=46	n=47	
**Overall SBP, median (IQR), mmHg**	97 (93,104)	97 (93,104)	97 (94,102)	101 (92,107)	97 (92,101)	.60
**Overall DBP, median (IQR), mmHg**	64 (61,67)	64 (62,67)	64 (60,67)	65 (60,68)	63 (62,66)	.66
**Overall MBP, median (IQR), mmHg**	75 (71,78)	75 (71,78)	75 (71,77)	76 (70,80)	74 (72,77)	.94
**Daytime SBP, median (IQR), mmHg**	102 (96,107)	102 (98,106)	102 (97,106)	106 (98,110)	101 (95,104)	.33
**Daytime DBP, median (IQR), mmHg**	68 (64,71)	69 (65,71)	68 (63,70)	68 (63,73)	66 (64,69)	.28
**Daytime MBP, median (IQR), mmHg**	78 (74,81)	79 (75,81)	77 (74,81)	80 (76,82)	78 (74,80)	.53
**Nocturnal SBP, median (IQR), mmHg**	90 (84,99)	89 (83,98)	90 (87,96)	93 (84,101)	91 (85,96)	.64
**Nocturnal DBP, median (IQR), mmHg**	57 (53,61)	56 (52,60)	57 (54,61)	59 (53,61)	58 (55,61)	.03
**Nocturnal MBP, median (IQR), mmHg**	69 (65,73)	67(64,71)	68 (66,73)	70 (64,74)	69 (65,73)	.15
**Drop in overall MBP, median (IQR)**	11.2 (7.59,15.5)	13.5 (8.00,18.2)	10.8 (8.28,15.1)	11.4 (8.67,15.2)	8.86 (6.58,13.5)	.01
**Non-dipper pattern, No (%)**	81 (43.3)	15 (31.9)	21 (44.7)	19 (41.3)	26 (55.3)	.04
**ABPM classification, No (%)**						.90
**Normal**	173 (95.6)	43 (95.6)	42 (93.3)	44 (97.8)	44 (95.7)	
**High-normal**	5 (2.76)	2 (4.44)	1 (2.22)	1 (2.22)	1 (2.17)	
**Hypertension**	3 (1.66)	0 (0)	2 (4.44)	0 (0)	1 (2.17)	

Frequencies and percentages have been obtained for categorical variables and median (IQR) for continuous variables. ABPM: ambulatory blood pressure monitoring; DBP: diastolic blood pressure; HB: hypoxic burden; IQR: interquartile range; MBP: mean blood pressure; SBP: systolic blood pressure.

© 2025 Mediano O et al. *JAMA Network Open*.

eTable 4. Measures of the Ambulatory 24-Hour Blood Pressure Parameters According to the Hypoxic Burden in Rapid Eye Movement Sleep

**ABPM parameters**	**HB - REM groups (based in quartiles)**				***P* for trend**
	**Quartile 1: ≤5.22**	**Quartile 2: (5.22; 14.1]**	**Quartile 3: (14.1; 29.55]**	**Quartile 4: >29.55**	
	**n=47**	**n=47**	**n=45**	**n=46**	
**Overall SBP, median (IQR), mmHg**	98 (93,105)	97 (93,104)	96 (92,102)	97 (93,104)	.35
**Overall DBP, median (IQR), mmHg**	64 (62,66)	65 (61,67)	64 (59,68)	63 (61,66)	.72
**Overall MBP, median (IQR), mmHg**	75 (71,78)	75 (71,79)	74 (70,78)	75 (71,78)	.72
**Daytime SBP, median (IQR), mmHg**	104 (99,110)	102 (97,107)	101 (95,105)	101 (94,108)	.06
**Daytime DBP, median (IQR), mmHg**	68 (65,71)	69 (64,72)	67 (62,71)	67 (63,71)	.18
**Daytime MBP, median (IQR), mmHg**	79 (77,83)	79 (75,82)	77 (72,81)	78 (74,81)	.19
**Nocturnal SBP, median (IQR), mmHg**	89 (83,99)	91 (86,99)	90 (86,96)	92 (85,100)	.39
**Nocturnal DBP, median (IQR), mmHg**	56 (52,59)	57 (53,61)	57 (54,61)	59 (55,62)	.008
**Nocturnal MBP, median (IQR), mmHg**	67 (63,72)	69 (66,73)	68 (65,73)	71 (65,74)	.05
**Drop in overall MBP, median (IQR)**	14.5 (9.28,18.2)	11.4 (7.70,15.4)	10.8 (7.59,13.6)	8.79 (6.16,12.9)	<0.001
**Non-dipper pattern, No (%)**	14 (29.8)	20 (42.6)	19 (42.2)	28 (60.9)	0.004
**ABPM classification, No (%)**					.77
**Normal**	43 (95.6)	42 (91.3)	44 (100)	42 (95.5)	
**High-normal**	2 (4.44)	2 (4.35)	0 (0)	1 (2.27)	
**Hypertension**	0 (0)	2 (4.35)	0 (0)	1 (2.27)	

Frequencies and percentages have been obtained for categorical variables and median (IQR) for continuous variables. ABPM: ambulatory blood pressure monitoring; DBP: diastolic blood pressure; HB: hypoxic burden; IQR: interquartile range; MBP: mean blood pressure; REM: rapid eye movement; SBP: systolic blood pressure.

© 2025 Mediano O et al. *JAMA Network Open*.

eTable 5. Measures of the Ambulatory 24-Hour Blood Pressure Parameters According to the Hypoxic Burden in Non–Rapid Eye Movement Sleep

ABPM parameters	HB - NREM groups (based in quartiles)				*P* for trend
	Quartile 1: ≤2.7	Quartile 2: (2.7; 6.5]	Quartile 3: (6.5; 17.6]	Quartile 4: >17.6	
	n=48	n=45	n=47	n=47	
**Overall SBP, median (IQR), mmHg**	97 (93,102)	98 (94,104)	100 (93,104)	97 (93,102)	.78
**Overall DBP, median (IQR), mmHg**	64 (61,67)	64 (60,67)	65 (60,68)	63 (62,66)	.99
**Overall MBP, median (IQR), mmHg**	74 (71,77)	75 (71,79)	76 (71,79)	74 (72,77)	.62
**Daytime SBP, median (IQR), mmHg**	100 (97,104)	102 (97,110)	103 (98,108)	101 (95,108)	.93
**Daytime DBP, median (IQR), mmHg**	69 (64,71)	68 (63,72)	67 (64,71)	68 (64,71)	.72
**Daytime MBP, median (IQR), mmHg**	77 (74,80)	78 (74,83)	79 (76,82)	78 (74,81)	.73
**Nocturnal SBP, median (IQR), mmHg**	90 (83,95)	91 (86,99)	92 (87,100)	91 (84,97)	.51
**Nocturnal DBP, median (IQR), mmHg**	56 (52,60)	56 (53,60)	58 (53,62)	58 (55,61)	.06
**Nocturnal MBP, median (IQR), mmHg**	68 (64,71)	67 (64,72)	70 (66,74)	69 (65,73)	.18
**Drop in overall MBP, median (IQR)**	11.4 (7.34,16.6)	13.2 (8.97,16.7)	10.3 (6.63,13.5)	9.76 (7.59,13.8)	.14
**Non-dipper pattern, No (%)**	19 (39.6)	16 (35.6)	22 (46.8)	24 (51.1)	.16
**ABPM classification, No (%)**					.94
**Normal**	43 (95.6)	41 (93.2)	46 (100)	43 (93.5)	
**High-normal**	2 (4.44)	1 (2.27)	0 (0)	2 (4.35)	
**Hypertension**	0 (0)	2 (4.55)	0 (0)	1 (2.17)	

Frequencies and percentages have been obtained for categorical variables and median (IQR) for continuous variables. ABPM: ambulatory blood pressure monitoring; DBP: diastolic blood pressure; HB: hypoxic burden; IQR: interquartile range; MBP: mean blood pressure; NREM: non rapid eye movement; SBP: systolic blood pressure.

© 2025 Mediano O et al. *JAMA Network Open*.

eTable 6. Measures of the Ambulatory 24-Hour Blood Pressure Parameters According to the Hypoxic Burden in the Supine Position

ABPM parameters	HB - supine groups (based in quartiles)				P for trend
	Quartile 1: ≤3.9	Quartile 2: (3.9; 10]	Quartile 3: (10; 27.05]	Quartile 4: >27.05	
	N=45	*N=42*	*N=41*	*N=44*	
**Overall SBP, median (IQR), mmHg**	97 (92,103)	97 (94,103)	101 (93,104)	97 (91,104)	.85
**Overall DBP, median (IQR), mmHg**	63 (60,67)	64 (61,67)	65 (61,67)	63 (61,66)	.82
**Overall MBP, median (IQR), mmHg**	74 (71,78)	75 (71,78)	76 (72,78)	74 (70,79)	.47
**Daytime SBP, median (IQR), mmHg**	102 (97,108)	101 (97,106)	104 (97,108)	101 (95,109)	.89
**Daytime DBP, median (IQR), mmHg**	68 (64,70)	68 (63,70)	68 (64,71)	66 (63,70)	.57
**Daytime MBP, median (IQR), mmHg**	78 (74,81)	77 (74,81)	79 (76,81)	78 (74,82)	.94
**Nocturnal SBP, median (IQR), mmHg**	90 (83,95)	91 (86,99)	91 (86,99)	92 (83,100)	.20
**Nocturnal DBP, median (IQR), mmHg**	56 (52,59)	56 (54,60)	57 (54,60)	58 (55,62)	.02
**Nocturnal MBP, median (IQR), mmHg**	68 (63,70)	68 (65,73)	69 (66,73)	71 (65,75)	.04
**Drop in overall MBP, median (IQR)**	13.5 (8.75,17.8)	11.8 (8.41,15.8)	10.3 (6.76,15.3)	9.72 (7.51,12.1)	.003
**Non-dipper pattern, No (%)**	14 (31.1)	17 (40.5)	19 (46.3)	24 (54.5)	.02
**ABPM classification, No (%)**					.77
**Normal**	41 (93.2)	39 (95.1)	40 (100)	40 (93.0)	
**High-normal**	2 (4.55)	1 (2.44)	0 (0)	2 (4.65)	
**Hypertension**	1 (2.27)	1 (2.44)	0 (0)	1 (2.33)	

Frequencies and percentages have been obtained for categorical variables and median (IQR) for continuous variables. ABPM: ambulatory blood pressure monitoring; DBP: diastolic blood pressure; HB: hypoxic burden; IQR: interquartile range; MBP: mean blood pressure; SBP: systolic blood pressure.

© 2025 Mediano O et al. *JAMA Network Open*.

eTable 7. Measures of the Ambulatory 24-Hour Blood Pressure Parameters According to the Hypoxic Burden in the Nonsupine Position

ABPM parameters	HB - non supine groups (based in quartiles)				*P* for trend
	Quartile 1: ≤3.2	Quartile 2: (3.2; 7.15]	Quartile 3: (7.15; 18.28]	Quartile 4: >18.28	
	n=50	n=43	n=46	n=48	
Overall SBP, median (IQR), mmHg	97 (94,104)	97 (93,104)	97 (91,104)	97 (93,102)	.47
Overall DBP, median (IQR), mmHg	65 (62,67)	64 (60,67)	62 (59,68)	64 (62,66)	.69
Overall MBP, median (IQR), mmHg	75 (72,78)	74 (71,78)	74 (70,78)	75 (73,78)	.94
Daytime SBP, median (IQR), mmHg	102 (99,107)	102 (97,106)	102 (95,108)	101 (95,106)	.22
Daytime DBP, median (IQR), mmHg	69 (65,71)	67 (64,69)	66 (62,72)	68 (64,71)	.31
Daytime MBP, median (IQR), mmHg	79 (76,81)	78 (74,81)	78 (72,82)	79 (75,81)	.61
Nocturnal SBP, median (IQR), mmHg	90 (83,99)	90 (86,96)	92 (84,98)	92 (85,99)	.54
Nocturnal DBP, median (IQR), mmHg	56 (52,60)	57 (54,60)	57 (53,61)	59 (56,62)	.02
Nocturnal MBP, median (IQR), mmHg	68 (64,71)	67 (66,73)	69 (64,73)	72 (66,74)	.09
Drop in overall MBP, median (IQR)	13.3 (8.44,18.2)	11.8 (7.50,15.1)	11.3 (9.03,13.9)	8.79 (6.39,13.2)	.008
Non-dipper pattern, n (%)	16 (32.0)	18 (41.9)	20 (43.5)	27 (56.2)	.02
ABPM classification, n (%)					.91
Normal	46 (95.8)	38 (92.7)	45 (97.8)	44 (95.7)	
High-normal	2 (4.17)	2 (4.88)	0 (0)	1 (2.17)	
Hypertension	0 (0)	1 (2.44)	1 (2.17)	1 (2.17)	

Frequencies and percentages have been obtained for categorical variables and median (IQR) for continuous variables. ABPM: ambulatory blood pressure monitoring; DBP: diastolic blood pressure; HB: hypoxic burden; IQR: interquartile range; MBP: mean blood pressure; SBP: systolic blood pressure.

© 2025 Mediano O et al. *JAMA Network Open*.

eFigure 1. Forest plot Showing the Result of the Multivariable Logistic Regression Analysis for Nondipper Pattern According to HB in REM Sleep

BMI: body mass index; CI: confidence interval; HB: hypoxic burden; REM: rapid eye movement. Adjusted for sex, age and BMI

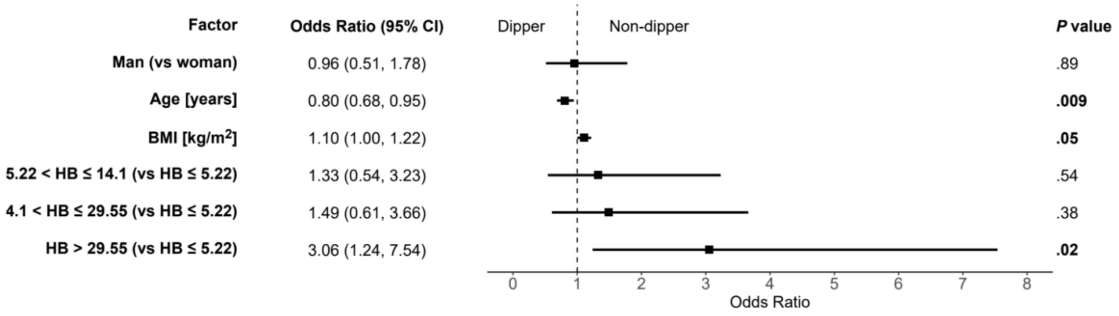

© 2025 Mediano O et al. *JAMA Network Open*.

eFigure 2. Forest plot Showing the Result of the Multivariable Logistic Regression Analysis for Nondipper Pattern According to HB in non-REM Sleep

BMI: body mass index; CI: confidence interval; HB: hypoxic burden; NREM: non-rapid eye movement Adjusted for sex, age and BMI

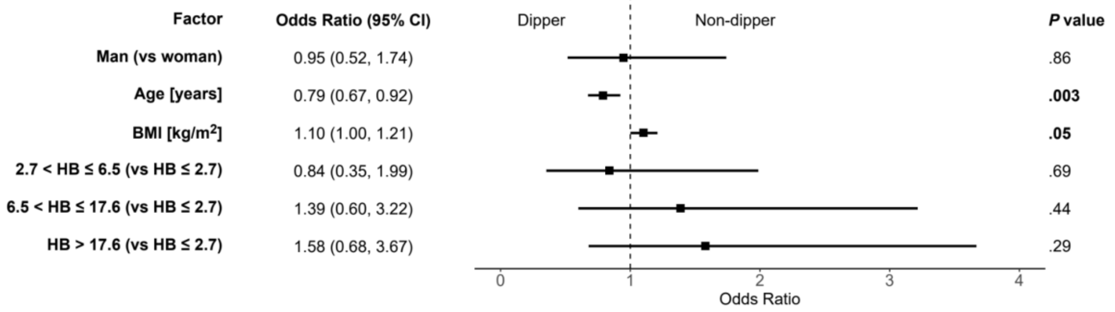

© 2025 Mediano O et al. *JAMA Network Open*.

eFigure 3. Forest Plot Showing the Result of the Multivariable Logistic Regression Analysis for Nondipper Pattern According to HB in Supine Position

BMI: body mass index; CI: confidence interval; HB: hypoxic burden Adjusted for sex, age and BMI

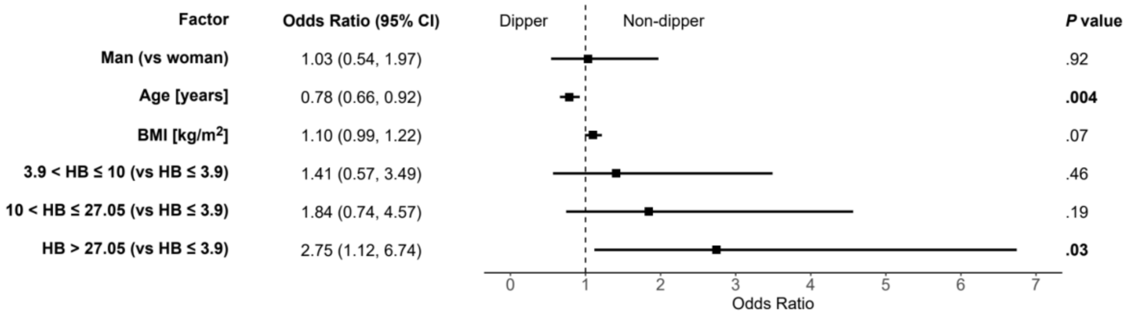

© 2025 Mediano O et al. *JAMA Network Open*.

eFigure 4. Forest Plot Showing the Result of the Multivariable Logistic Regression Analysis for Nondipper Pattern According to HB in Nonsupine Position

BMI: body mass index; CI: confidence interval; HB: hypoxic burden Adjusted for sex, age and BMI

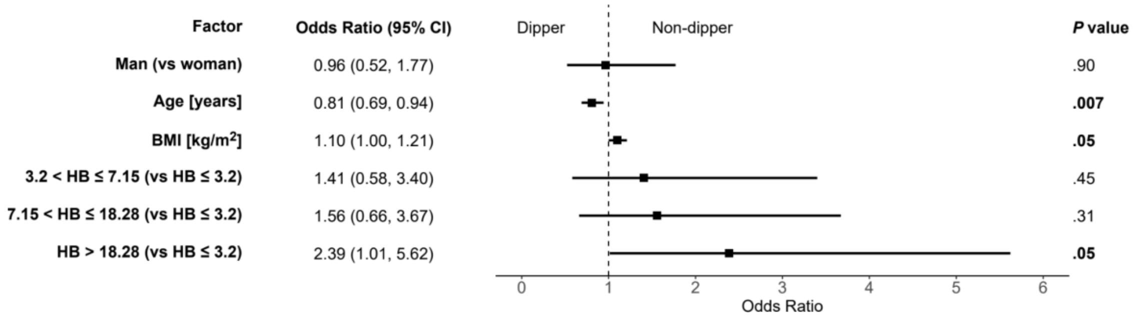

© 2025 Mediano O et al. *JAMA Network Open*.
