# Supplementary material for: Cardiovascular Risk Through Hypoxic Burden in Children With Sleep Apnea: A Secondary Analysis of a Nonrandomized Clinical Trial
Source: JAMA Netw Open. 2025 Oct 23;8(10):e2538744. doi: 10.1001/jamanetworkopen.2025.38744 (PMC12550637; doi:10.1001/jamanetworkopen.2025.38744)
Supplement: Supplement 3. — Data Sharing Statement [file jamanetwopen-e2538744-s003.pdf]

Data Sharing Statement

Mediano. Cardiovascular Risk Through Hypoxic Burden in Children With Sleep Apnea. *JAMA Netw Open*. Published October 23, 2025. doi:10.1001/jamanetworkopen.2025.38744

Data
Additional Information: NCT03696654 clinicaltrials.gov
Data available: Yes
Data types: Other (please specify)
Additional Information: Data would be available on request and subject to approval by the steering committee.
How to access data: Data would be available on request and subject to approval by the steering committee.
When available: With publication

Supporting Documents
Document types: None

Additional Information
Who can access the data: Data would be available on request and subject to approval by the steering committee.
Types of analyses: Data would be available on request and subject to approval by the steering committee.
Mechanisms of data availability: Data would be available on request and subject to approval by the steering committee.
Any additional restrictions: None
